# Fos regulates age-dependent neuroinflammation in a *VAP^P58S^* model of amyotrophic lateral sclerosis

**DOI:** 10.1242/dmm.052810

**Published:** 2026-07-24

**Authors:** Namrata Pramod Kulkarni, Aparna Thulasidharan, Amarendranath Soory, Pulkit Goel, Sanhita Sarkar, Vidyadheesh Kelkar, Girish S. Ratnaparkhi

**Affiliations:** Department of Biology, Indian Institute of Science Education and Research, Pune 411008, India

**Keywords:** Amyotrophic lateral sclerosis, Vesicle-associated membrane protein-associated protein B, CRISPR/Cas9, Neuroinflammation, Kayak

## Abstract

Amyotrophic lateral sclerosis (ALS) is a fatal neurodegenerative disorder characterized by progressive loss of motor function. Here, we developed a *Drosophila* model of ALS8 (*VAPB^P58S^*) using CRISPR/Cas9 genome editing. VAPB is an endoplasmic reticulum-based adapter protein associated with and regulating intracellular membrane:membrane contact sites. *VAPB^P58S^* flies showed progressive age-dependent motor deficits and a shortened lifespan, paralleling features of the human disease. *VAPB^P58S^* brains exhibited age-dependent neuroinflammation, as measured by whole-transcriptome quantitative mRNA sequencing, suggesting a broad, low-grade enhancement of signalling across multiple immune pathways (Toll, Imd, Jak-STAT and c-Jun). Our results indicated that glial cells in the brain are the site of brain inflammation and identified the *Drosophila* orthologue of Fos (Kayak) as a key modulator of age-dependent inflammation. In accordance, we found that overexpression of wild-type *kayak* or its dominant-active variant *kayak^K357R^* in glia reduced inflammation and, concomitantly, improved motor function. In contrast, knockdown of glial *kayak* accelerated age-dependent deterioration of motor function and enhanced neuroinflammation. Our study underscores the roles of glial-modulated brain inflammation in dictating ALS8 progression and identifies *kayak* as a central negative regulator of neuroinflammation in disease.

## Teaser

**Summary:** In a *Drosophila* model, we uncover definitive evidence of age-dependent neuroinflammation, originating from glial cells and regulated by Fos, as a key mechanism underlying amyotrophic lateral sclerosis-8.

## INTRODUCTION

Amyotrophic lateral sclerosis (ALS) is a disease characterized by muscle atrophy due to the degeneration of neurons in the cortex, brainstem and spinal cord (Feldman et al., 2022; Mitchell and Borasio, 2007; Pasinelli and Brown, 2006). The disease starts with mild symptoms such as weakness, difficulty swallowing and slurred speech, but quickly progresses to more severe symptoms such as motor dysfunction, laboured breathing and paralysis. Death typically occurs due to respiratory failure within 3-5 years of diagnosis (Boylan, 2015; Cleveland and Rothstein, 2001; Tartaglia et al., 2007). Ninety percent of ALS cases are sporadic ALS with no identified family history, whereas the remaining 10% are classified as familial ALS, involving one or more affected family members (Boylan, 2015; Marin et al., 2012). More than 30 loci contributing to distinct or overlapping pathomechanisms – such as oxidative stress, neuroinflammation, lipid dysregulation, mitochondrial dysfunction and proteostasis failure – have been associated with ALS, indicating the multifactorial and complex nature of the disease (Abel et al., 2012; Jiang et al., 2024; Robberecht, 2000; Tendulkar et al., 2022; Thulasidharan et al., 2024).

Although ALS was first described almost a century ago, little has been achieved in terms of treatment, with very few available drugs, offering only modest benefits (Paganoni et al., 2020; Wijesekera and Leigh, 2009; Yoshino and Kimura, 2006). Additionally, owing to its oligogenic nature, the molecular mechanism(s) underlying ALS remains poorly understood, rendering the disease largely untreatable. Thus, efforts to investigate molecular mechanisms and gene networks underlying ALS in model organisms can pave the way for the development of more effective therapies.

The eighth ALS locus to be identified was vesicle-associated membrane protein (VAMP) associated protein B (*VAPB*). A dominant proline-to-serine point mutation at position 56 (P56S) in the major sperm protein (MSP) domain was identified in Portuguese-Brazilian families and is associated with familial ALS (Nishimura et al., 2004). *VAPB* is a definitive ALS locus (Abel et al., 2012; McCann et al., 2020). It encodes a single-pass type IV integral membrane protein that resides on the endoplasmic reticulum (ER) membrane. VAPB has three domains: the C-terminal transmembrane domain, which is inserted into the ER membrane; a coiled-coil domain that helps form multimers; and the N-terminal MSP domain, which faces the cytosol (Borgese et al., 2021a; James and Kehlenbach, 2021; Kors et al., 2022). The MSP domain can interact with a wide range of partner proteins that decorate the surface of organelle membranes and facilitate the formation of the membrane-membrane contact site (MCS) between the ER and these (other) organelles. VAPB, through the MCS, plays a crucial role in efficiently coordinating inter-organelle processes, thereby maintaining cellular homeostasis. These cellular processes include membrane trafficking, lipid transport and lipid homeostasis, immune signalling, calcium signalling, autophagy, stress response and protein homeostasis (reviewed in Borgese et al., 2021a; James and Kehlenbach, 2021; Murphy and Levine, 2016; Neefjes and Cabukusta, 2021; Reinisch et al., 2025; Zhao et al., 2018). Modulation and disruption of VAP-related MCSs can affect these functions, in whole or in part (Kodama et al., 2025; Obara et al., 2024; Stoica et al., 2014).

Our laboratory has been interested in studying the *Drosophila* orthologue of *VAPB*, *Vap33/CG5014* (hereafter referred to as *VAP*), as a model for amyotrophic lateral sclerosis-8 [ALS8; Online Mendelian Inheritance in Man (OMIM) 608627], which is caused by heterozygous mutation in the *VAPB* gene (OMIM 605704) on chromosome 20q13. As a first step, we used an enhancer-suppressor screen to identify the VAP gene regulatory network in flies. The study uncovered multiple ALS-orthologue loci, such as *Superoxide dismutase 1* (*Sod1*), *Alsin* (also known as *Als2*), *TAR DNA-binding protein 43* (*TDP43*; also known as *TBPH*), and *Target of rapamycin* (*TOR*; also known as *mTor*), as genetic interactors with *VAP* (Deivasigamani et al., 2014). Subsequently, we studied the effects of Sod1 and TOR modulation on VAP^P58S^ aggregates; *VAP^P58S^* is the *Drosophila* equivalent of *VAPB^P56S^*. Notably, our study revealed that, in the larval brain, reducing either Sod1 levels or TOR signalling increased cellular reactive oxygen species (ROS) levels, which, in turn, enhanced clearance of VAP^P58S^ aggregates by triggering the ubiquitin-proteasome system (UPS). Further, using a null-rescue model developed by the Tsuda laboratory, *gVAP^P58S^* (Moustaqim-Barrette et al., 2014), we discovered that autophagy, rather than UPS, was the dominant mechanism contributing to aggregate clearance (Thulasidharan et al., 2024) in the adult brain. Additionally, we found that age-dependent inflammation was a key factor in determining the extent of disease progression, with Immune deficiency (Imd) signalling upregulated in *gVAP^P58S^* (Tendulkar et al., 2022).

In the current study, we aimed to extend our understanding of the roles of neuroinflammation in disease pathogenesis, using a CRISPR/Cas9 genome-edited *VAP^P58S^* model generated in our laboratory. As a first step, we conducted an age-dependent quantitative transcriptomic experiment on the heads of the CRISPR-generated *VAP^P58S^* mutant line. Interestingly, the *VAP^P58S^* transcriptome showed a low-grade increase in the levels of inflammatory markers with age. Intriguingly, our analysis revealed that the neuroinflammatory changes were global rather than restricted to a single pathway, with inflammatory markers detected across the Imd, Toll, c-Jun N-terminal kinase (JNK) and Janus kinase (Jak)-Signal transducer and activator of transcription (STAT) pathways. We found that, in *VAP^P58S^* flies, glial expression of the *Drosophila* orthologue of Fos, *kayak* (*kay*), reduced transcription of inflammatory markers and, concomitantly, slowed the progression of age-dependent motor defects. Our study highlights the role of Kay as a broad, critical modulator of neuroinflammation and disease progression and functionally links VAP, located in membrane:membrane contact sites to signalling by innate immune pathways.

## RESULTS

### A genome-edited *VAP^P58S^* model of ALS8

The initial fly *VAP^P58S^* disease models relied on overexpression (OE) of the transgene (Han et al., 2012; Mitne-Neto et al., 2011; Pennetta et al., 2002; Ratnaparkhi et al., 2008; Tsuda et al., 2008) and subsequent assessment of neuronal physiology. Two weaknesses of the OE model were that the mutant VAP^P58S^ was present in excess, and endogenous VAP^WT^ (wild-type control) was present. Subsequently, Hiroshi Tsuda's laboratory developed an improved model in which an endogenous *gVAP* (*gVAP^WT^* or *gVAP^P58S^*) insert on the third chromosome was used to rescue the lethality of the VAP null (*ΔVAP*) (Moustaqim-Barrette et al., 2014). The Tsuda disease model, *ΔVAP;gVAP^P58S^*, unlike its control *ΔVAP;gVAP^WT^*, has a shorter lifespan and shows progressive motor dysfunction (Moustaqim-Barrette et al., 2014). The *ΔVAP;gVAP^P58S^* model has VAP^P58S^ inclusions in the brain, with cells showing ER expansion and ER stress (Moustaqim-Barrette et al., 2014; Tendulkar et al., 2022; Thulasidharan et al., 2024). The Tsuda model did not exhibit the artefacts observed in the OE models, although it was constrained to studying only males. Additionally, two chromosomes (I and III) were occupied, limiting complex genetic manipulations.

**Fig. 1.**
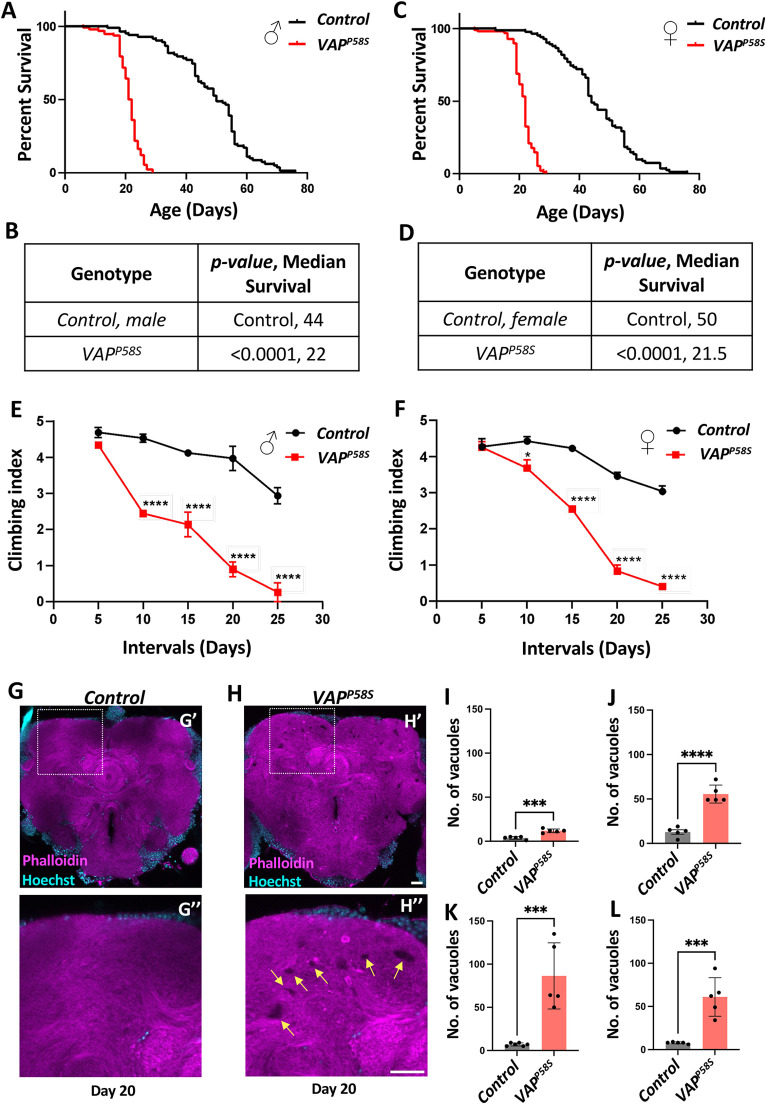
**The *VAP^P58S^* mutant has reduced survival compared to that of *VAP^WT^* and exhibits progressive age-dependent motor defects.** (A) Lifespan curves for *VAP^P58S^* males (red curve) and *VAP^WT^* (black curve). *n*=80-100 flies for every genotype, *N*=3. Curve comparison was done using log-rank test. The combined *P*-value for the set is *P*<0.0001. (B) Tabulated results for the lifespan results in A, quantified in terms of median lifespan (ML). (C) Lifespan curve for *VAP^P58S^* females (red curve) and *VAP^WT^* (black curve). *n*=80-100 flies for every genotype, *N*=3. Curve comparison was done using log-rank test. The combined *P*-value for the set is *P*<0.0001. (D) Tabulated results for the lifespan results in C, quantified in terms of ML. (E) Climbing index for *VAP^P58S^* males (red curve) plotted for intervals 5, 10, 15, 20 and 25 days. *VAP^WT^* (black curve) was used as the control. *n*=25-30, *N*=3 flies for every genotype. Day 5, *P*>0.05 (not significant); day 10, ****P<0.0001; day 15, ****P<0.0001; day 20, ****P<0.0001; day 25, ****P<0.0001. (F) Climbing index for *VAP^P58S^* females (red curve) plotted for intervals 5, 10, 15, 20 and 25. *VAP^WT^* (black curve) was used as the control. *n*=25-30, *N*=3 flies for every genotype. Day 5, *P*>0.05 (not significant); day 10, *P<0.05; day 15, ****P<0.0001; day 20, ****P<0.0001; day 25, ****P<0.0001. (G-H″) Single sections of confocal images showing nuclear stain Hoechst (cyan) and F-actin stain Phalloidin (magenta). 20-day-old brains of control (*CS*; G) and *VAP^P58S^* (H) flies are shown*.* Insets (G″,H″) are 3× magnified images of dotted line boxes in G′ and H′, respectively. Arrows point to pathological vacuoles. Scale bars: 20 μm. (I-L) Histograms of the total numbers of vacuoles in the central brain of 5-day-old *CS* (*n*=5, 3.800±1.643) and *VAP^P58S^* (*n*=5, 11.80±2.168) flies, *P*=0.0002 (I); 10-day-old *CS* (*n*=5, 12.80±5.541) and *VAP^P58S^* (*n*=5, 55.60±10.14) flies, *P*<0.0001 (J); 15-day-old *CS* (*n*=6, 7.0±1.789) and *VAP^P58S^* (*n*=5, 86.40±38.29) flies, *P*=0.0006 (K); and 20-day-old *CS* (*n*=5, 7.800±1.095) and *VAP^P58S^* (*n*=5, 61.00±22.29) flies, *P*=0.0007 (L). Statistical significance was calculated using unpaired two-tailed *t*-test. Bar and error bars denote mean and s.d. ***P<0.001, ****P<0.0001.

Here, we generated a *VAP^P58S^* mutant line by editing the wild-type *VAP* locus using CRISPR/Cas9 genome editing (Fig. S1). Details of the editing experiment are provided in the Materials and Methods section, with a summary included in Fig. S1. The *VAP^P58S^* genome-edited line (*VAP^P58S^* hereafter) is homozygous viable with a lifespan comparable to that of *ΔVAP;gVAP^P58S^*. We found that both *VAP^P58S^* mutant males (Fig. 1A, red curve) and females (Fig. 1C, red curve) showed a significant reduction in survival compared to that of the controls (*VAP^WT^*; Fig. 1A,C, black curve). *VAP^P58S^* males had a median lifespan (ML) of 22 days, while females showed a ML of 21.5 days, which was significantly less than the MLs of 44 and 50 days for *VAP^WT^* males and females (Fig. 1B,D), respectively. Compared with *VAP^P58S^*, the *ΔVAP;gVAP^P58S^* males had an ML of 27 days. The *VAP^WT^* animals used were ‘CRISPR’ controls – flies that had been part of the genome-editing pipeline but did not acquire the P58S mutation in the *VAP* locus.

### *VAP^P58S^* mutant flies show a progressive increase in age-dependent motor defects

To characterize motor defects in the *VAP^P58S^* line, we performed an age-dependent startle-induced negative geotaxis assay (Madabattula et al., 2015; Tendulkar et al., 2022). The *VAP^P58S^* mutant flies, both males and females, showed no obvious motor defects at eclosion. With age, both male and female *VAP^P58S^* flies showed deterioration in climbing ability. The climbing assay was performed on days 5, 10, 15, 20 and 25 for both sexes. *VAP^P58S^* mutant males showed no motor defects on day 5 compared to *VAP^WT^*, but showed a significant decline in the climbing index by day 10, which further worsened with age. The *VAP^WT^* males showed a mild decline in motor abilities as age progressed (Fig. 1E).

Similarly, *VAP^P58S^* mutant females showed no motor defects on day 5 compared to age-matched *VAP^WT^* females, but showed a progressive deterioration in motor function with age. *VAP^WT^* females showed a mild decline in motor abilities with age (Fig. 1F). Both male and female *VAP^P58S^* mutant lines became completely immobile by day 25.

We evaluated neurodegenerative progression in *VAP^P58S^* animals using multiple markers. The Tsuda disease model (Moustaqim-Barrette et al., 2014) showed vacuoles in the brain and highlighted upregulation of ER stress pathways. Our laboratory has also demonstrated ER stress and VAP aggregates (Tendulkar et al., 2022; Thulasidharan et al., 2024) in ageing flies for the same model. For the *VAP^P58S^* animals, we measured age-dependent changes in vacuoles in the adult brain (Fig. 1G,H). We found an increase in the number (Fig. 1H,I-L; Fig. S2B) and size (Fig. S2B,C-F) of vacuoles compared to those in controls (Fig. 1G,I-L; Fig. S2A,C-F). We used anti-BiP (also known as Hsc70-3) antibody staining (Thulasidharan et al., 2024) to demonstrate ER stress (Fig. S2G-I) in *VAP^P58S^*. We also observed VAP aggregates (Chaplot et al., 2019; Thulasidharan et al., 2024) in the *VAP^P58S^* line (Fig. S2J-N). VAP aggregate density, as shown earlier (Thulasidharan et al., 2024), is stable with age, while BiP puncta decline with age (Thulasidharan et al., 2024).

Thus, we conclude that *VAP^P58S^* mutant flies exhibit progressive, age-dependent motor defects and neurodegeneration in the brain, and that their behavioural phenotypes are comparable to those observed in the existing *ΔVAP;gVAP^P58S^* disease model (Moustaqim-Barrette et al., 2014).

### *VAP^P58S^* flies show enhanced age-dependent neuroinflammation

**Fig. 2.**
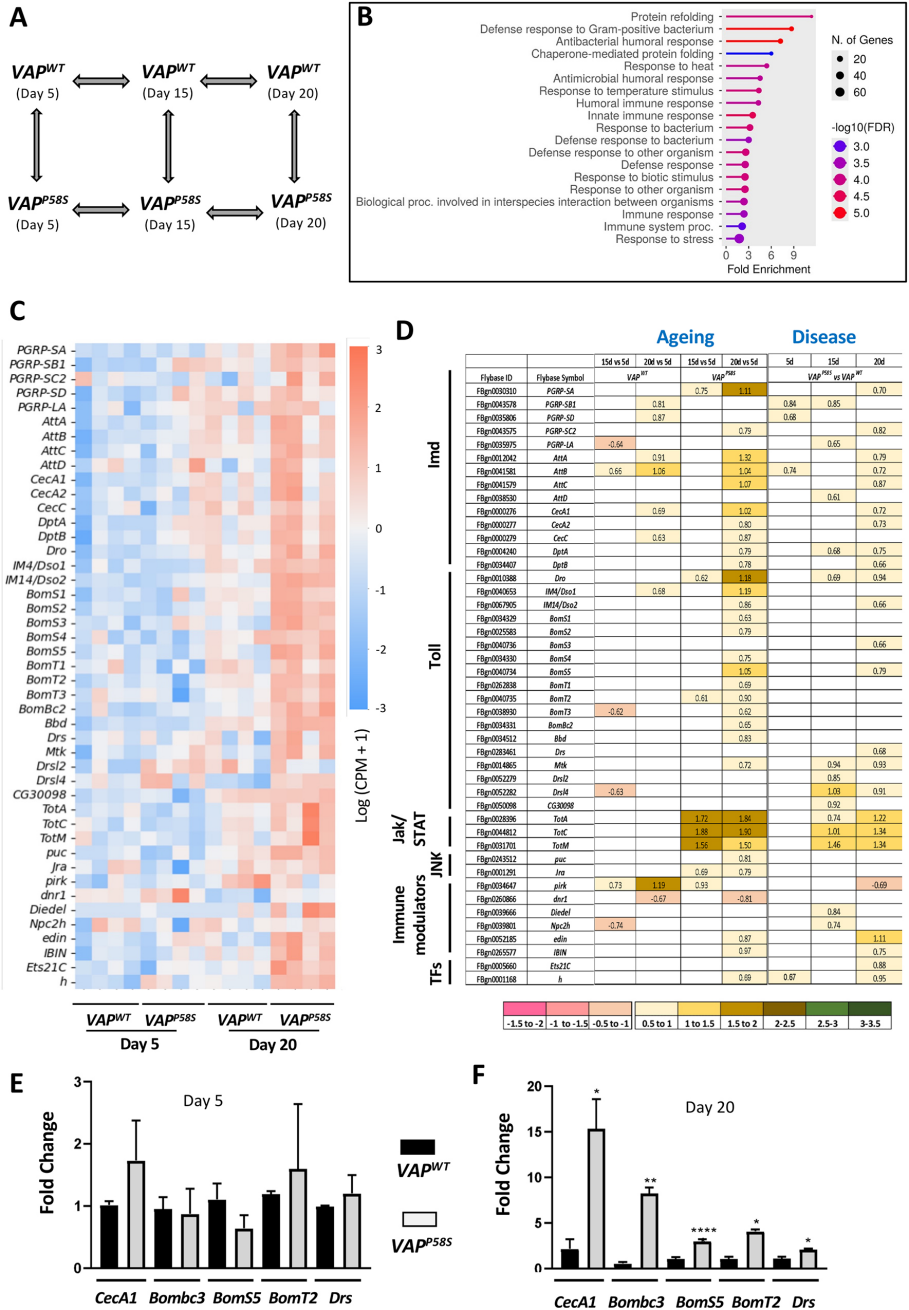
**Transcriptomics on male *VAP^P58S^* heads showing increased neuroinflammation with age.** (A) Genotypes and timepoints used for the 3′ mRNA sequencing and the pairwise comparison done to obtain differentially expressed genes. (B) Gene Ontology (GO) enrichment analysis for significantly differentially expressed genes between *VAP^P58S^* and *VAP^WT^* at days 5, 15 and 20 combined. FDR, false discovery rate. (C) Heatmap showing the normalized expression counts for immune genes significantly differentially expressed between *VAP^P58S^* and *VAP^WT^* for days 5 and 20. CPM, counts per million. (D) Tabular representation of log_2_ fold change (log_2_FC) values for immune gene transcripts. The genes have been categorized into their immune pathway or the molecular function they perform. ‘Ageing’ depicts a comparison of the genotype with itself as it ages, while ‘Disease’ shows a comparison of *VAP^P58S^* with *VAP^WT^* at age-matched timepoints. d, day. (E,F) qRT-PCR for the Imd pathway target *CecA1* and Toll pathway targets *Bombc3*, *BomS5*, *BomT2* and *Drs* at days 5 (E) and 20 (F), for *VAP^WT^* and *VAP^P58S^* heads. The *y*-axis shows log_2_FC normalized to the housekeeping gene *rp49*. Values shown as mean+s.e.m. *n* (number of heads)=40-50, *N* (biological replicates)=3. Statistical analysis was done using two-way ANOVA. Multiple comparisons were performed using Tukey's test. *P<0.05, **P<0.01, ****P<0.0001.

In a previous study (Tendulkar et al., 2022) using the *ΔVAP;gVAP^P58S^* model of ALS8, we uncovered a role for the IMD-nuclear factor kappa beta (NFκB) pathway in disease progression, with Caspar OE in glia reducing neuroinflammation and improving motor function. In this study, to thoroughly quantify the onset of inflammation in the adult brain, we measured the head transcriptome in ageing *VAP^P58S^* mutant flies, with *VAP^WT^* animals used as age-matched controls. Transcriptomes were measured at days 5, 15 and 20 in the heads of *VAP^P58S^* and *VAP^WT^* males, based on lifespan and motor-function data (Fig. 2). The *VAP^P58S^* line enabled us to collect data from both males and females (Figs S3-S5), allowing us to address sexual dimorphism in our disease model.

For females, data were collected at two time points, day 5 and day 20. Our experiments utilized the Lexogen QuantSeq kit to generate multiplexed libraries for measuring quantitative gene expression (described by Soory and Ratnaparkhi, 2022; see Materials and Methods). A schematic of the experimental timepoints, genotypes and pairwise comparisons is shown in Fig. 2A and Fig. S4A, with Fig. S3A showing the 3′ mRNA-sequencing workflow. Volcano plots for male and female data, with a few important genes highlighted, have also been plotted (Fig. S3B,C, respectively). Fig. 2 displays data for males. Gene Ontology (GO) analysis (Fig. 2B) of the differentially expressed genes between *VAP^P58S^* and *VAP^WT^* males indicated that the most significant changes in the transcriptome are associated with host defence response, with genes classified as ‘immunity’ markers upregulated. The immune response category is followed by a ‘Chaperones/protein refolding’ category that includes Heat shock proteins (Hsps) of the Hsp70 and Hsp60 families, as well as small Hsps, which are biomarkers of ageing (Tower, 2011). Both categories are expected, as research from our laboratory has highlighted both protein aggregation (Chaplot et al., 2019; Thulasidharan et al., 2024) and inflammation (Tendulkar et al., 2022) as key features of the *ΔVAP;gVAP^P58S^* model (Moustaqim-Barrette et al., 2014). What is surprising, however, is the dominance and preponderance of these genes in the transcriptomic landscape of the disease model. Of the ∼500 genes statistically upregulated in the *VAP^P58S^* model, ∼10% are immune genes, and ∼2.5% are protein-folding related. Genes linked to neuronal function are also modulated, as discussed in a subsequent section.

To understand how the transcriptome changes across genotypes and with age, we plotted a heatmap for a subset of these differentially expressed genes (Fig. 2C). The immune genes, especially ‘defence genes’ showed increased expression in *VAP^P58S^* compared to that in *VAP^WT^*, as well as age-dependent enhancement (15-day data excluded for clarity in Fig. S3D). The heat map depicts normalized expression counts for immune genes, indicating weak to moderate changes in transcript levels. Nevertheless, the twofold to sixfold increase in transcripts of defence genes at day 20 is striking, especially given that *VAP^P58S^* at day 5 shows non-significant changes compared to age-matched *VAP^WT^*.

To further gain insights into the immune pathways contributing to this low-grade inflammation, we segregated defence genes into known immune signal transduction pathways and tabulated log_2_ fold change (log_2_FC) values for each transcript (Fig. 2D). A large number of defence genes represented are anti-microbial peptide (AMP) genes that are strong indicators of upregulation of specific immune-responsive signalling pathways. We categorized the genes that changed as ‘Ageing’ versus ‘Disease’. For ageing, we highlighted genes that showed a significant change in expression levels at 20 or 15 days versus 5 days, for both *VAP^P58S^* and *VAP^WT^*, independently. For disease, we compared age-matched *VAP^P58S^* versus *VAP^WT^* heads (5-day, 15-day or 20-day).

In the ‘Ageing’ context, both *VAP^WT^* and *VAP^P58S^* showed an increase in the expression of immune genes; however, *VAP^P58S^* showed higher log_2_FC values than *VAP^WT^*, for a subset of genes (Fig. 2D). Transcripts with higher values included Attacins (Atts), Cecropins (Cecs), Diptericins (Dpts), *Drosocin* (*Dro*), Daishos (Dsos), Bomanins (Boms), *Metchnikowin* (*Mtk*), Turandots (Tots) and *puckered* (*puc*). *Jun-related antigen* (*Jra*), the transcription factor downstream of JNK signalling, was also upregulated. The corresponding immune pathways for each gene are marked (*y*-axis) in Fig. 2D. In the ‘Disease’ context, many defence genes were found to be upregulated in *VAP^P58S^* at day 15 and day 20. Transcripts with higher values included Tots, Atts, Cecs and Boms among others*.* Interestingly, transcripts of the peptidoglycan recognition proteins were mildly upregulated, while *pirk*, a negative regulator of immune signalling, was downregulated (Fig. 2D). We selected a few AMPs and, in an independent experiment, performed quantitative reverse transcription PCR (qRT-PCR) on *Drosophila* heads. Data for 5- and 20-day-old *VAP^WT^* and *VAP^P58S^* flies are shown in Fig. 2E,F, respectively. The results were comparable to those from 3′ mRNA sequencing, with 5-day-old *VAP^P58S^* flies showing no change in AMP levels and 20-day-old *VAP^P58S^* flies showing significantly elevated AMP levels compared those of *VAP^WT^*.

In agreement with the male data, *VAP^P58S^* females also showed a low-grade increase in immune-responsive gene transcripts, with age. GO analysis showed terms such as ‘regulation of antifungal innate immune response’, ‘innate immune response’ and ‘humoral immune response’ among others (Fig. S4B). A heatmap showing the significantly differentially expressed genes indicated elevated levels of defence genes (Fig. S4C), in agreement with the male transcriptome. As observed for the male data (Fig. 2), the genes were mapped to ‘Ageing’ and ‘Disease’ (Fig. S4D). AMP transcripts across Toll, Imd and Jak-STAT pathways showed a weak upswing in transcripts with age, compared to those in *VAP^WT^*. When the male and female datasets were compared, females had higher log_2_FC values overall, especially for ageing. qRT-PCR data for 5-day-old and 20-day-old flies (Fig. S4E,F, respectively), for a few transcripts confirm the trend in the transcriptomics data.

### *VAP^P58S^* flies show age-dependent changes in neuronal function

**Fig. 3.**
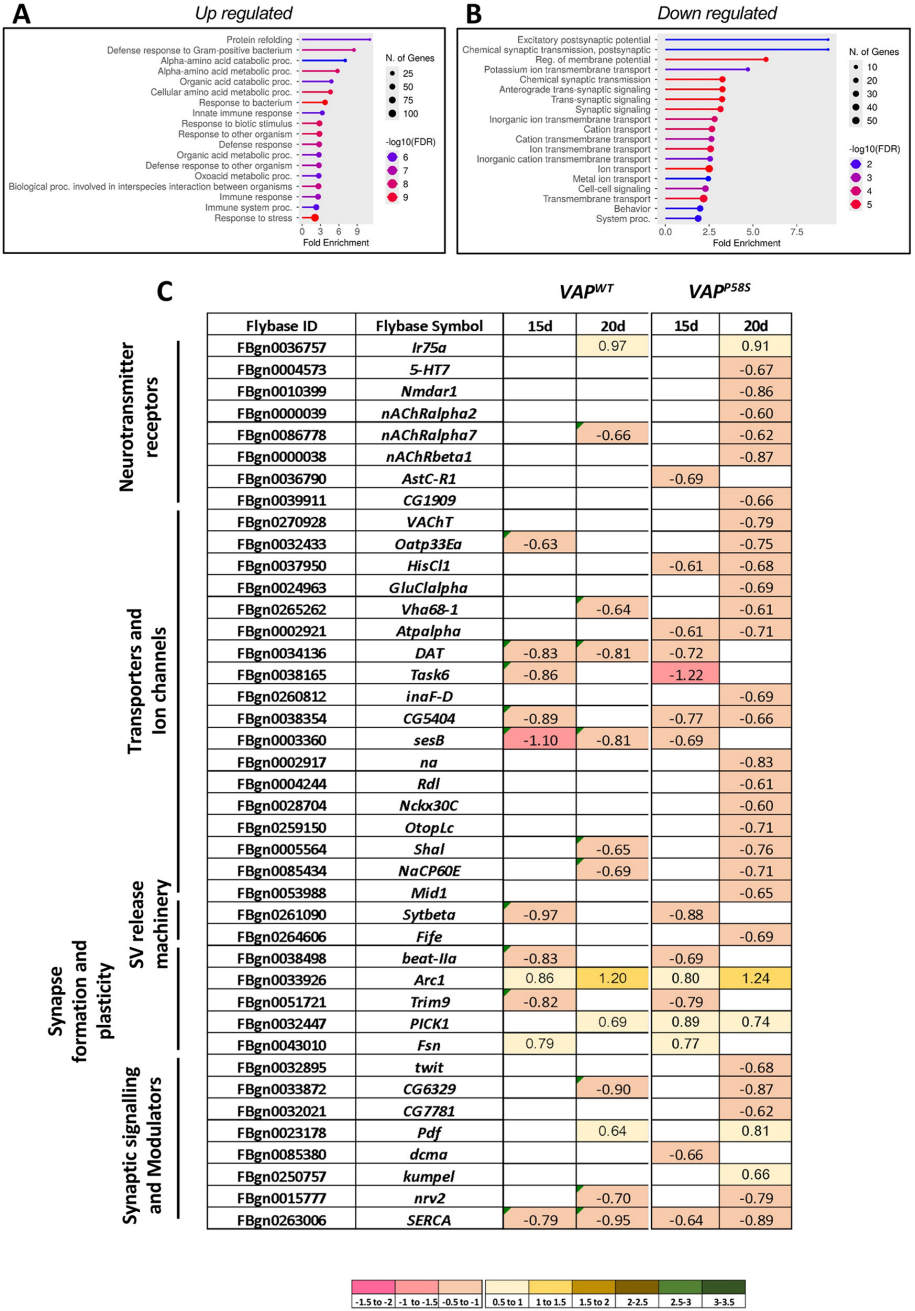
**Transcriptomic analysis showing age-dependent changes in synaptic signalling in *VAP^P58S^* males.** (A) GO analysis of significantly upregulated genes in *VAP^P58S^* between day 5 and days 15/20. Fig. S5 displays the heat map and volcano plots for these datasets, while Fig. S6 displays data for females. (B) GO analysis of significantly downregulated genes in *VAP^P58S^* relative to those in *VAP^WT^* between day 5 and days 15/20. (C) Tabular representation of log_2_FC values for synaptic gene transcripts. The genes have been categorized based on the functions they perform. The table shows comparison of *VAP^P58S^* at days 15 and day 20 with *VAP^P58S^* at day 5. The same comparison has been done for *VAP^WT^*. The colour table depicts log_2_FC values. Negative values indicate downregulation, and positive values indicate upregulation.

As *VAP^P58S^* animals showed age-dependent motor deficits, we also examined changes in transcripts for genes involved in neuronal function (Fig. 3; Figs S5 and S6). GO analysis of the differentially expressed genes for *VAP^P58S^* (20 and 15 day, normalized to day 5) suggested that, in addition to upregulation of immune response genes, genes involved in categories such as ‘Synaptic transmission’, ‘Membrane potential’ and ‘Inorganic ion transport’ were downregulated in both male and female *VAP^P58S^* flies with age (Fig. 3A,B; Fig. S6A,B). The genes were classified as within the ‘Neurotransmitter receptors’, ‘Transporters and ion channels’, ‘Synaptic vesicle release machinery’, Synapse formation and plasticity’ and ‘Synaptic Signalling and modulators’ categories. Log_2_FC values were tabulated for both *VAP^P58S^* and *VAP^WT^* (Fig. 3C). Although the overall trend was for a mild decrease in transcripts with age, the reduction was more substantial in *VAP^P58S^* than in *VAP^WT^* and affected a larger subset of genes. This suggested overall acceleration of the decline in levels of *VAP^P58S^* neuronal transcripts in comparison to those in *VAP^WT^*, indicating enhanced ageing.

In accordance, *VAP^P58S^* females also showed downregulation of most neuronal genes with age compared to those in *VAP^WT^* (Fig. S6C). Heatmaps and volcano plots for males and females are shown in Fig. S5A,B and Fig. S6D,E, respectively.

Our observations in the *VAP^P58S^* transcriptome align well with findings from multiple independent head transcriptomic studies evaluating ageing and age-related disorders in *Drosophila* (Kounatidis et al., 2017; Girardot et al., 2006; Katzenberger et al., 2013; Hanson and Macdonald, 2025). These transcriptomes show downregulation of transcripts associated with synaptic transmission at various levels. The neural gene categories listed above have been repeatedly enriched in these studies. Broad classes of neurotransmitter receptors such as cholinergic nicotinic acetylcholine receptors, glutamate receptors and NMDAR have been shown to be downregulated with age. This decline is consistent with the age-associated dampening of synaptic responsiveness. Additionally, downregulation of genes involved in regulating excitability Na^+^/K^+^/Cl^−^ ion channels such as *Na^+^ pump α-subunit* (*Atpα*) and *stress-sensitive B* (*sesB*) in these brain transcriptomes points towards impaired electrical signalling (Pacifico et al., 2018). Moreover, downregulation of Shaker cognate I (ShaI) protein levels is known to be associated with age-dependent motor decline in flies (Vallejos et al., 2021). Apart from these, components of the synaptic vesicle formation and release machinery including Synaptotagmins (Syts) show decreased expression with age, reflecting weakening of the synapse and loss of synaptic plasticity (Pacifico et al., 2018).

Neuropeptides such as *Pigment-dispersing factor* (*Pdf*), which is involved in maintaining normal circadian rhythm, similarly show an increase in expression, indicating changes in broader circuit neuromodulation (Umezaki et al., 2012). Overall, the coordinated downregulation of these receptors, ion channels, transporters, and vesicle release and structural synapse-associated genes points to disruption of synaptic structure and function as flies age. Additionally, the ageing transcriptomes in these various studies show increased expression of immune-associated genes.

Our transcriptomic analysis of *VAP^P58S^* flies, thus, reveals age-associated immune dysregulation and synaptic dysfunction in both male and female *VAP^P58S^* mutants. This further underscores inflammation as a key pathological mechanism in disease progression. Additionally, our study supports the idea that the *VAP^P58S^* flies undergo accelerated or premature ageing marked by loss of neuronal gene expression and heightened proteostasis stress.

### *VAP^P58S^* gut does not show inflammatory changes

In addition to our transcriptomic experiments on the heads of the *VAP^P58S^* mutants, we performed age-dependent transcriptomics on the gut. Our goal was to uncover significant transcriptomic changes in the gut of *VAP^P58S^*, both in the context of the gut:brain axis and effects of *VAP^P58S^* on ageing and disease within the gut. Unlike in the brain, the gut does not show large changes in gene expression in either the ‘ageing’ or the ‘disease’ context (Fig. S7). A volcano plot for the gut differentially expressed genes (Fig. S7A) between *VAP^P58S^* and *VAP^WT^* highlights the few genes that show change. GO analysis indicated that these genes are involved in disaccharide metabolic process, oogenesis, sexual reproduction and cell development. Unlike in the head transcriptome, we could not identify trends that associated with defence response or immunity (Fig. S7B). Tabulation of the differentially expressed genes between *VAP^P58S^* and *VAP^WT^* at 5 days and 20 days (Fig. S7C) shows very few immune genes (Fig. S7D). These changes do not indicate a significant role for the gut:brain axis in the *VAP^P58S^* model. The experiments were first carried out in females, and because the *VAP^P58S^* gut did not show significant changes, we did not generate data for the male gut transcriptome.

In conclusion, data from the gut transcriptomics of *VAP^P58S^* show minimal changes compared to *VAP^WT^*, suggesting that neuroinflammation in the *VAP^P58S^* adult brain is not correlated with changes in the gut transcriptome.

### A glial enhancer/suppressor screen identifies Kay as a key regulator of motor function in *VAP^P58S^* flies

Previous work from our laboratory using the *ΔVAP;gVAP^P58S^* model identified the Imd pathway as a regulator of glial inflammation (Tendulkar et al., 2022). In this study, we screened genes in the Toll and JNK pathways to establish their roles in the ALS8 context. As in the earlier study (Tendulkar et al., 2022), we balanced *reverse polarity* (*repo*)*-Gal4* with *VAP^P58S^* to establish a *VAP^P58S^; repoGal4; +* line*. VAP^P58S^; repoGal4* females were crossed to either knockdown (KD), OE, dominant-negative (DN) or constitutive active (CA) *UAS* lines of *dorsal* (*dl*), *Dorsal-like immune factor* (*Dif*), *Cactus* (*Cact*), *spatzle* (*spz*), *spatzle 2* (*spz2*; also known as *NT1*), *spatzle 5* (*spz5*), *spatzle 6* (*spz6*) and *Toll10b* (also known as *Tl*) for the Toll pathway or *kay*, *Jra*, *basket* (*bsk*) and *hemipterous* (*hep*) for the JNK pathway. F1 males were selected, with climbing ability being used as the readout (Fig. S8A). Gene modulations that worsened the climbing ability of the *VAP^P58S^* flies were termed as ‘Enhancers’, whereas those that improved the climbing ability were considered as ‘Suppressors’ (Fig. S8B).

The first pathway we examined was the Toll pathway. Fig. S8C depicts results from climbing assays performed on days 5, 10 and 15. KD of *dl* (*P*>0.05, 2, light-blue curve), *Dif* (L1; *P*>0.05, 3, light green), *Dif* (L2; *P*>0.05, 4, dark-green curve), *Cact* (*P*>0.05, 5, pink), *spz2* (*P*>0.05, 8, yellow curve), *spz5* (*P*>0.05, 9, orange curve), *spz6* (*P*>0.05, 10, dark-blue curve) did not result in any significant change in the climbing index in *VAP^P58S^* compared to the age-matched control *VAP^P58S^; repoGal4* (Fig. S8C; 1, black curve). OE of the active form of the classical Toll receptor, *Toll10b*, similarly showed no change (Fig. S8C; *P*>0.05, 11, grey curve). *Cact* OE showed mild suppression of motor defects at day 10 (Fig. S8C; *P*<0.05, 6, purple curve). Similarly, *spz* KD mildly suppressed motor defects at day 10 (Fig. S8C; *P*<0.05, 7, brown curve). Fig. S8D provides a tabulated summary with colour-coded *P*-values. Shades from light pink to magenta indicate increasing levels of motor deterioration (Enhancers), whereas shades from yellow to dark green indicate improvement in motor function (Suppressors).

The second pathway that we examined was the JNK pathway. Fig. S8E shows climbing assays for the modulation of JNK pathway genes. KD of *kay* resulted in significant enhancement of the motor function defect in the *VAP^P58S^* flies at day 15 (Fig. S8E; *P*<0.001, 2, red curve), whereas *kay* OE improved climbing ability at day 10 (Fig. S8E; *P*<0.001, 4, light-green curve). OE of *Fra.Fbz* (L1), the dominant-negative form of *kay*, resulted in no change in climbing index (Fig. S8E; *P*>0.05, 3, dark-pink curve), while OE of *Fra.Fbz* (L2) was lethal. OE of *Jra^DN^* (Fig. S8E; *P*<0.01, 7, blue curve) as well as KD of *bsk* (Fig. S8E; *P*<0.05, 8, sea green curve) resulted in mild enhancement of motor defects at day 15. OE of *bsk^DN^*, on the contrary, resulted in very mild suppression (Fig. S8E; *P*<0.05, 9, orange curve). *Jra* KD (Fig. S8E; *P*>0.05, 5, ochre curve) and OE of either *Jra* (Fig. S8E; *P*>0.05, 6, pink curve), *bsk* Fig. S8E; (*P*>0.05, 10, purple curve) or *hep* (Fig. S8E; *P*>0.05, 11, grey curve) did not affect the climbing index. OE of the constitutively active *hep* was lethal. A summary table presents the *P*-values for different intervals, colour coded as previously described (Fig. S8F).

Our glial screen, thus, indicated that the JNK pathway is involved in regulating motor function in *VAP^P58S^*, and we focused subsequent experiments on understanding the glial role of *kay* in the disease model in greater detail.

### *kay*/*kay^SCR^* OE in glia partially rescues motor function

Because the glial enhancer/suppressor screen identified *kay* as a critical modulator of motor function in *VAP^P58S^* flies, we generated additional *kay* lines to validate the rescue. Of the two variants, one expresses wild-type *kay* (*UAS-kay*) and the second expresses the small ubiquitin-like modifier (SUMO) conjugation resistant (SCR) form of *kay* (*UAS-kay^K357R^*; further referred to as *UAS-kay^SCR^*). In mammals, SCR Fos is resistant to SUMO conjugation and is a more active form (Bossis et al., 2005). Hence, we included it in our OE experiments as an alternative allele, with the expectation that it would give similar or stronger rescue of motor function.

**Fig. 4.**
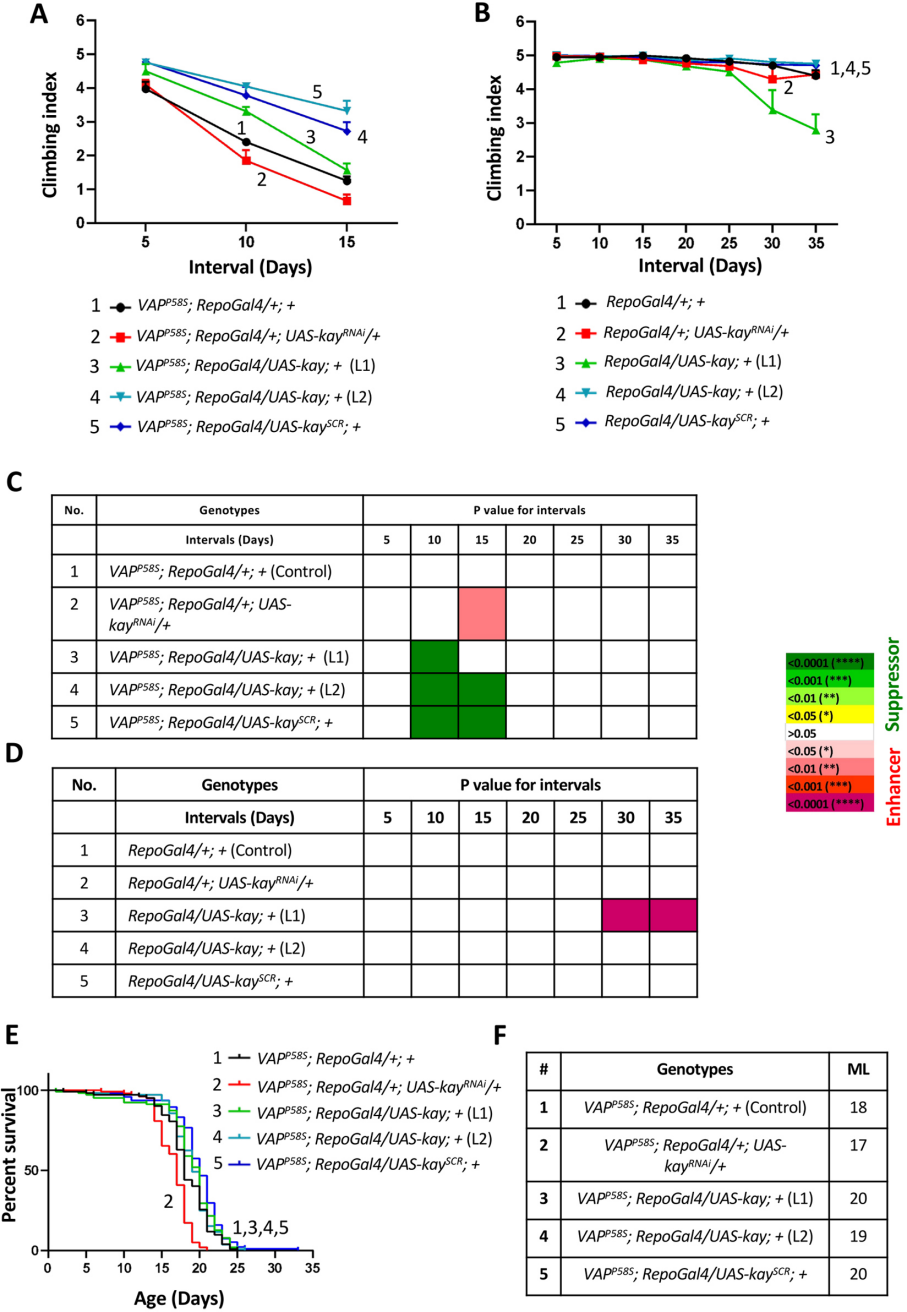
**Glial overexpression (OE) of *kay* or *kay^SCR^* partially rescues motor function and lifespan of the disease model.** (A) Climbing indices for glial knockdown (KD) of *kay* (2, red), *kay* OE (L1; 3, light green), *kay* OE (L2; 4, light blue), *kay^SCR^* (5, dark blue) in the background of *VAP^P58S^*. *VAP^P58S^; repoGal4/+; +* males were used as age-matched controls (1, black). Values shown as mean±s.e.m. *n*=25-30, *N*=3. (B) Climbing indices for glial KD of *kay* (2, red), *kay* OE (L1; 3, light green), *kay* OE (L2; 4, light blue) and *kay^SCR^* (5, dark blue) in the background of wild type. *repoGal4/+; +* males were used as age-matched controls (1, black). Values depicted as mean±s.e.m. *n*=25-30, *N*=3. (C,D) Summary table for the gene modulations in A (C) and B (D). The individual *P-*values have been colour coded. Statistical analysis as previously described. (E) Lifespan curves for glial KD of *kay* (2, red), *kay* OE (L1; 4, light green), *kay* OE (L2; 4, light blue) and *kay^SCR^* (5, dark blue) in the background of *VAP^P58S^*. *VAP^P58S^*;*repoGal4/+; +* males were used as controls (1, black). The combined *P*-value for the set is *P*<0.0001. *n*=80-100, *N*=3. (F) Results tabulated in terms of ML for each genotype.

Fig. 4A shows the climbing index curve for glial modulation of *kay* in the background of *VAP^P58S^*, with Fig. 4C tabulating enhancement/suppression of *VAP^P58S^* function based on *P*-value. KD of *kay* in the glia showed significant worsening of motor function defects in the *VAP^P58S^* flies by day 15 (Fig. 4A; *P*<0.01, 2, red curve) compared to *VAP^P58S^; repoGal4/+; +* control (Fig. 4A; *P*<0.01, 1, black curve). OE of *kay*, with the line procured from Bloomington *Drosophila* Stock Center (BDSC; L1) showed significant rescue of motor function at day 10 (Fig. 4A; *P*<0.0001, 3, green curve). OE of *kay* (L2) using the laboratory generated line as well as the *kay^SCR^* line resulted in stronger rescue of motor function at day 10 and day 15 (Fig. 4A; *P*<0.0001, 4 and 5, light-blue and dark-blue curves, respectively).

Next, we looked at the effect of all the modulations on motor function in wild-type flies (Fig. 4B,D). Flies overexpressing *kay* showed deterioration of motor function from day 30 onwards (Fig. 4B; *P*<0.0001, 3, green curve), which was in contrast to the rescue of motor function observed in the *VAP^P58S^* flies. *repoGal4/+; +* males were used as controls (Fig. 4B; 1, black curve).

**Fig. 5.**
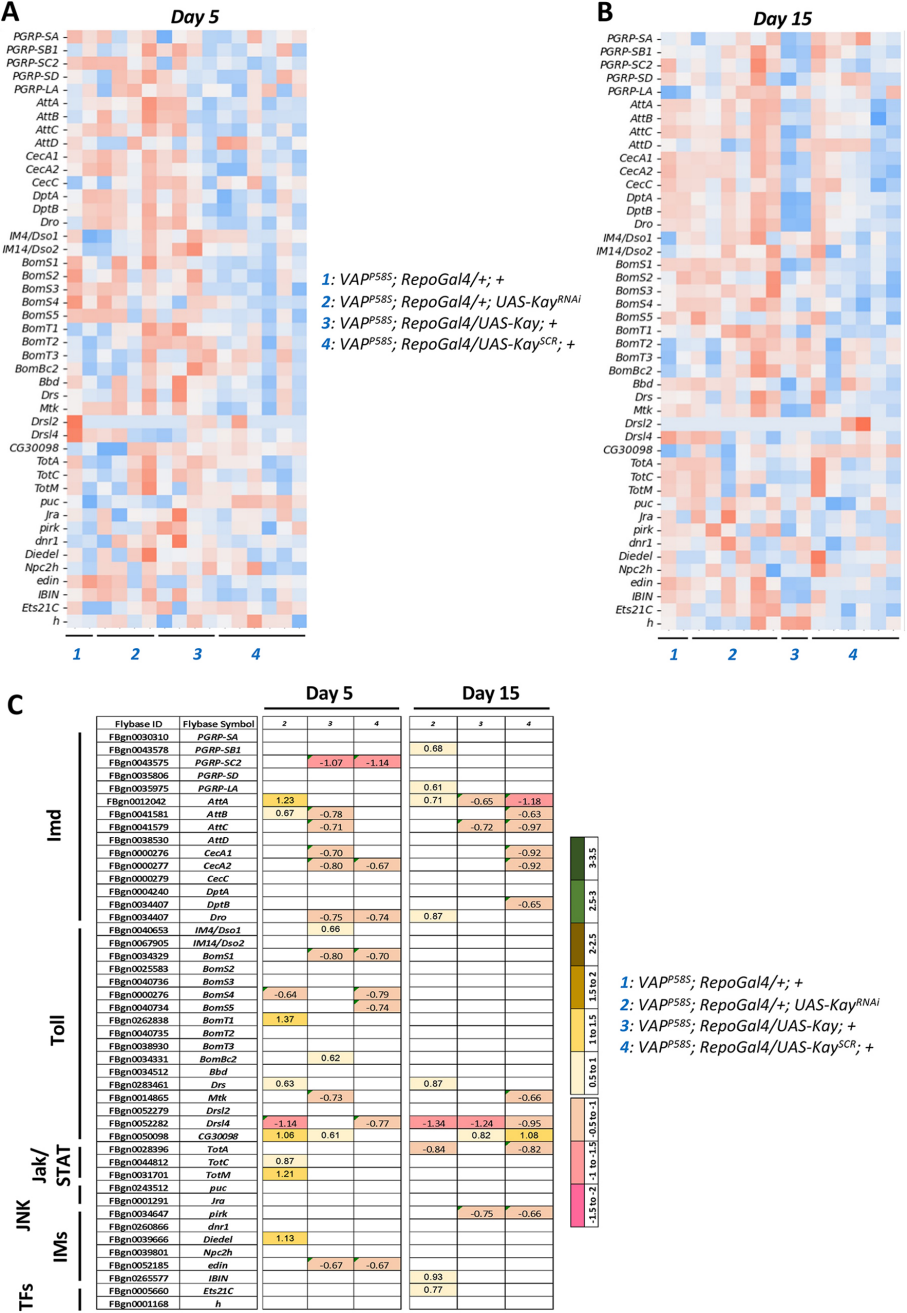
***kay* or *kay^SCR^* in glia rescues neuroinflammation in *VAP^P58S^* males.** (A) Heatmap showing the normalized expression counts for immune genes significantly differentially expressed in 2, *VAP^P58S^; repoGal4/+; UAS-Kay^RNAi^*; 3, *VAP^P58S^; repoGal4/UAS-Kay*; 4, *VAP^P58S^; repoGal4/UAS-Kay^SCR^*; with 1, *VAP^P58S^; repoGal4/+* being used as the control (at day 5). (B) Heatmap showing the normalized expression counts for immune genes significantly differentially expressed in 2, *VAP^P58S^; repoGal4/+;UAS-Kay^RNAi^*; 3, *VAP^P58S^; repoGal4/UAS-Kay*; 4, *VAP^P58S^; repoGal4/UAS-Kay^SCR^*; with 1, *VAP^P58S^; repoGal4/+* being used as the control (at day 5). (C) Log_2_FC values plotted for significantly differentially expressed immune genes for day 5 and day 15. The colour codes and genotypes are shown on the side.

*VAP^P58S^* flies exhibited reduced lifespan, in addition to motor defects. As *kay* KD worsened motor function in these flies and *kay/kay^SCR^* rescued it, we examined whether lifespan showed a similar trend. *VAP^P58S^; repoGal4/+; +*, which were used as controls, showed an ML of 18 days (Fig. 4E,F, 1, black curve). KD of *kay* showed very mild reduction of lifespan, with the ML being 17 days (Fig. 4E,F, 2, red curve). OE of *kay* (L1), *kay* (L2) and *kay^SCR^* resulted in mild increase in lifespan, with MLs being 20, 19 and 20 days (Fig. 4E,F, 3, 4 and 5, green, light-blue and dark-blue curves), respectively.

Thus, our experiments established that *kay* acts as a modifier of ALS8-like symptoms in the *Drosophila VAP*^*P58S*^ model, with OE in glia leading to improvement in the motor deficit of *VAP^P58S^* males.

### Glial *kay/kay^SCR^* OE mitigates age-dependent neuroinflammation

*VAP^P58S^* flies show age-dependent neuroinflammation in the head. Further, glial knockdown of *kay* exacerbated age-dependent motor deterioration in *VAP^P58S^* mutants, whereas its OE suppressed these motor defects. This would suggest that motor function in the *VAP^P58S^* model was a function of the extent of inflammation. We therefore overexpressed and knocked down *kay* in the head and examined changes in the progressive low-grade inflammation observed in the disease model.

Fig. 5 displays the set of immune genes identified as upregulated in *VAP^P58S^* mutant males; many of these were defence genes, visualized using their normalized expression profiles across four genotypes (1, *VAP^P58S^; repoGal4/+*; 2, *VAP^P58S^; repoGal4/+;UAS-kay^RNAi^*; 3, *VAP^P58S^; repoGal4/UAS-kay*; 4, *VAP^P58S^; repoGal4/UAS-kay^SCR^*) and two timepoints. The heatmaps for day 5 (Fig. 5A) and day 15 (Fig. 5B) indicate differences in immune gene expression across the four genotypes. Upon *kay* KD, the expression of immune genes was elevated compared to that in age-matched controls. In contrast, overexpressing *kay* or *kay^SCR^* reduced immune gene expression at both 5 and 15 days (Fig. 5A,B, respectively). Intriguingly, the reduction in immune gene expression obtained with OE of *kay^SCR^* was better than, or on par with, that seen with *kay^WT^*. This outcome reinforces previous conclusions from mammalian studies that SCR Fos is an active form (in this case, a stronger inhibitor of immune gene expression). To quantify gene changes, we plotted the log_2_FC values for these immune genes. Most AMPs from Imd, Toll and Jak-STAT pathways were found to be elevated upon *kay* KD in the glia at both timepoints. However, glial OE of *kay* or *kay^SCR^* downregulated the expression of the AMPs. The strongest reduction was seen in *AttA*. Additionally, *PGRP-SC2* showed a significant downregulation of expression. Other AMPs – such as *AttB*, *AttC*, *CecA1*, *CecA2*, *DptB*, *BomS4*, *BomS5*, *Mtk* and *TotA* – also showed milder reduction in expression (Fig. 5C).

Is Kay a known regulator of defence genes, and does the literature indicate a potential mechanism for Kay-modulated transcriptional repression? *kay* was one of the 45 genes (Fisher et al., 2023) modulated in the embryo to study their transcriptional targets during embryogenesis (10-12 h and also 16-18 h post-fertilisation). Fisher et al. (2023) found that *kay* primarily regulated 25 defence response genes, including Boms, showing ∼30% overlap with defence genes identified in our study (Fig. S9A-C). Importantly, all these genes were negatively regulated by Kay, with *kay* KD leading to their upregulation. Further, we analysed chromatin immunoprecipitation sequencing (ChIP-seq) data from the Encyclopedia of Regulatory Networks (modERN) consortium and mapped Kay occupancy to the gene bodies of all genes in *Drosophila.* We found enriched peaks for Kay binding on the promoters of 4762 genes (Fig. S9D, Clusters 2, 4 and 5). However, the Kay-responsive genes identified in our study did not appear to have binding sites for Kay, suggesting that Kay acts indirectly, possibly regulating transcription via another transcription factor. In the few cases in which Kay showed enriched binding to promoters of genes, such as *Rel*, *dl* and *Stat92E*, our mRNA-sequencing data showed no change in the expression of these genes upon *kay* modulation (Fig. S9E).

Our transcriptomic data, thus, suggest that OE of *kay* or *kay^SCR^* contributes to significant rescue of climbing ability in the *VAP^P58S^* mutant by clearing neuroinflammation. We believe that this is achieved via Kay's broad repression of major immune pathways. However, the absence of promoter binding and the lack of changes in the expression levels of the major immune transcription factors suggest an indirect regulatory mechanism by Kay.

## DISCUSSION

ALS is a neuronal disease primarily affecting motor neurons. The health of neurons, however, also depends on neighbouring glial cells, which perform a host of metabolic, synaptic and immune functions (Allen and Lyons, 2018; Bittern et al., 2021; Bonvento and Bolaños, 2021; Fields and Stevens-Graham, 2002; Tsacopoulos and Magistretti, 1996; Volkenhoff et al., 2015). Glia shape the inflammatory environment in the brain, and regulated levels of inflammation are neuroprotective (Linnerbauer et al., 2020; Shi and Yong, 2025).

Chronic or persistent neuroinflammation is a pathological hallmark of most neurodegenerative disorders, including ALS (Johnson and Lukens, 2025; Thonhoff et al., 2018; Zhang et al., 2023; Zhao et al., 2013). Although ALS was initially considered a disease primarily associated with motor neuron degeneration, the involvement of non-neuronal cells such as glia is increasingly being explored and established (Boillée et al., 2006). Mounting evidence points to a glia-derived pro-inflammatory state in various animal models (Koehn et al., 2025; O'Rourke et al., 2016; Yamanaka et al., 2008) and in human patients with ALS (Femiano et al., 2024; Gagliardi et al., 2024; Mishra et al., 2017; Steinacker et al., 2018).

Glial neuroinflammation has emerged as a central non-cell-autonomous mechanism in ALS. In neurodegeneration, microglia shift to a chronically activated, pro-inflammatory phenotype driven by signals such as misfolded proteins, damage-associated molecular patterns (DAMPs) and cytokines (Chiu et al., 2008; Weydt et al., 2004; Beers et al., 2006; Martens et al., 2022). Activated microglia release tumour necrosis factor (TNF-α; also known as TNF), interleukin (IL)-1β, IL-6, ROS and nitric oxide, and activate the NOD-like receptor pyrin domain-containing protein 3 (NLRP3) inflammasome, amplifying local inflammation and promoting motor-neuron vulnerability (Deora et al., 2020; Nagai et al., 2007; Xu et al., 2025). These reactive astrocytes, termed ‘A1 astrocytes’ (Liddelow et al., 2017), reduce metabolic and synaptic support to neurons. Further, peripheral immune cells infiltrate the central nervous system, compromising the blood-brain barrier (Beers et al., 2008; Henkel et al., 2004). Neurons, glia and secreted pro-inflammatory factors create a chronic immune response that accelerates motor-neuron degeneration and disease progression in ALS. *Drosophila* models of ALS also support the central role of glial ‘activation’, their phagocytic roles, demonstrating activation of Toll/Imd or JNK pathways in the brain in neurodegeneration or traumatic brain injury models (Cao et al., 2013; Elguero et al., 2023; Katzenberger et al., 2013; Kounatidis et al., 2017; Lee et al., 2020; Li et al., 2018; Tendulkar et al., 2022; van Alphen et al., 2022).

In our *Drosophila VAP^P58S^* model of ALS, we observe an increase in low-grade inflammation as animals age and lose motor function. The progressive decline in motor function correlates with increasing inflammation. A targeted enhancer/suppressor motor function screen identified the JNK transcription factor Kay as a key disease modifier. Kay, along with its partner Jra, forms a heterodimeric Activator protein (AP)-1 complex (Kockel et al., 2001; Perkins et al., 1990) implicated in diverse physiological pathways, including stress response (Alfonso-Gonzalez and Riesgo-Escovar, 2018; Biteau et al., 2011; Kockel et al., 2001; Perkins et al., 1990; Tafesh-Edwards and Eleftherianos, 2020). The AP-1 complex (Fos/Jun in vertebrates) is assumed to be the most stable form, although both Kay and Jra can function as homodimers (Alfonso-Gonzalez and Riesgo-Escovar, 2018). JNK signalling, with Kay/Jra as transcriptional regulators, is a major regulator of the immune response (Kleino and Silverman, 2014; Park et al., 2004; Silverman et al., 2003; Tafesh-Edwards and Eleftherianos, 2020). Both Jra and Kay are expressed in neurons and glia, and their vertebrate orthologues have been implicated in neurodegenerative diseases (Gogia et al., 2020; Sahana et al., 2023).

We found that KD of *kay* in the glia led to a worsening of motor function, while OE of *kay* or *kay^SCR^* improved motor function in *VAP^P58S^* mutant lines. Kay appears to function as a broad regulator of the basal immune response, affecting defence genes downstream of multiple immune pathways. As Kay is a transcriptional regulator, and is negatively regulating defence genes, one possible mechanism is for it to bind to promoters of defence genes and function as a dampening factor; however, our analysis appears to rule this out as a primary mechanism.

What then is the link between VAP, an ER-associated protein, with Kay? How does this relationship, in glial cells, regulate age-dependent progression of gene-regulatory networks, especially those related to innate immune pathways? How does a point mutation in VAP lead to accelerated ageing and inflammation? A hint comes from studies in viral immunity (Ji et al., 2025), which links VAPB in mammals to innate immune signalling via the cyclic guanosine 5′­monophosphate (GMP)-adenosine 5′­monophosphate (AMP) synthase (cGAS)-stimulator of interferon genes (STING) pathway (Decout et al., 2021; Dvorkin et al., 2024). The cGAS-STING pathway responds to aberrant cytosolic DNA and is an evolutionarily conserved mechanism for defence against microorganisms (Marques et al., 2024). Ji et al. (2025) found that VAPB binds to and sequesters the ER-resident STING under basal conditions. In response to cytoplasmic double-stranded DNA (dsDNA) stimulation, STING translocates away from the ER, presumably to the Golgi or peroxisomes, and this perinuclear translocation triggers an inflammatory response. The authors implicate TANK-binding kinase 1 (TBK1) as a central facilitator of the VAPB/cGAS-STING pathway, which phosphorylates and activates downstream transcriptional regulators. VAPB thus acts as a negative regulator of the STING-associated immune response (Ji et al., 2025). TBK1 is one of a host of cytoplasmic kinases (CKs) that function as crucial hubs in signaling, acting as molecular switches that phosphorylate targets to regulate transcriptional activation and repression. The CKs integrate signals, forming complexes with receptors, and are essential for both initiating and fine-tuning innate and adaptive immunity. The CKs are associated with cellular compartments, including the plasma membrane, the Golgi, endosomes and the ER. CKs can also travel to the nucleus as part of their activation. In *Drosophila*, CKs such as Pelle, Hopscotch and TGF-β-activated kinase 1 (Tak1) are upstream of the major immune pathways and fine-tune the expression of defence genes. IκB kinase epsilon (IKKε) is the fly orthologue of TBK1 and functions to regulate the degradation of Death-associated protein inhibitor of apoptosis 1 (Diap1), a central regulator of apoptotic signalling. STING is also conserved in flies (Liegeois and Ferrandon, 2022), with Martin et al. (2018) demonstrating a role for *Drosophila melanogaster* STING in the immune response. Martin et al. (2018) found that STING associates with cyclic dinucleotides and utilizes the Imd pathway to activate defence genes. Intriguingly, Tak1, a MAP3K kinase related to TBK1, is a key regulator of NFκB signalling in *Drosophila* and is upstream of both Rel (Silverman et al., 2003; Vidal et al., 2001) and JNK (Geuking et al., 2005; Igaki et al., 2002) pathways.

**Fig. 6.**
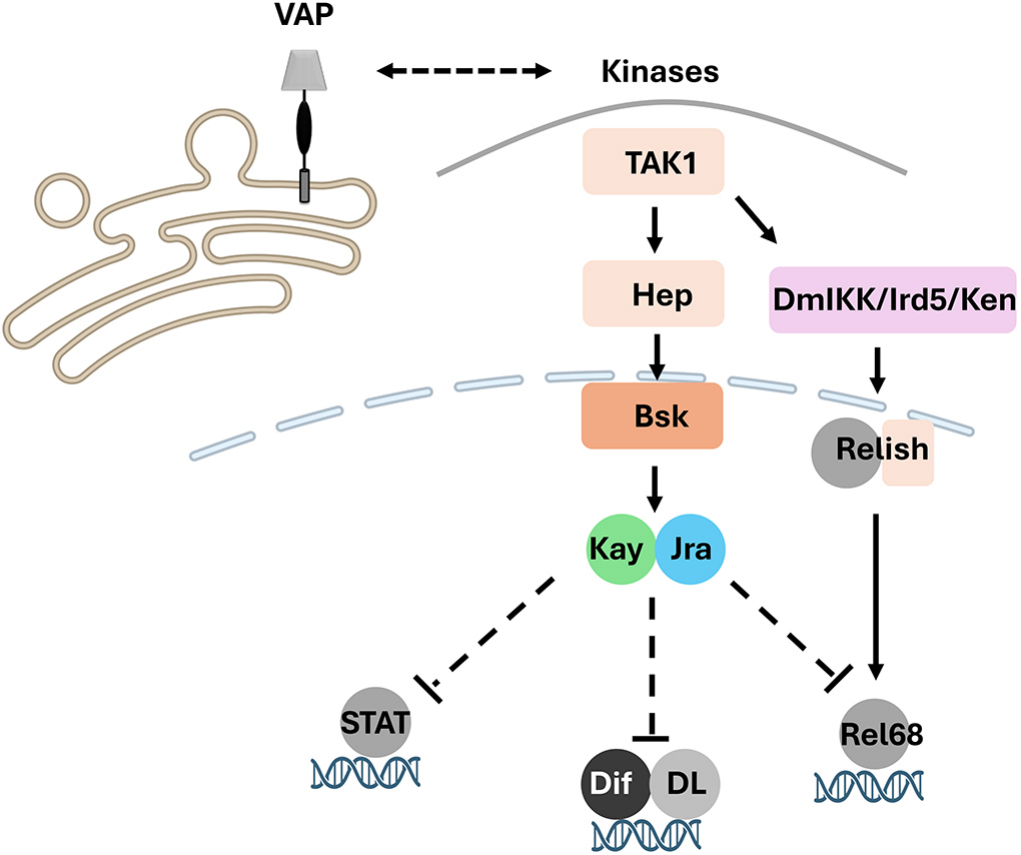
**Model for regulation of the glial immune response.** *VAP^P58S^* flies show age-dependent low-grade enhancement of neuroinflammation, evident from the increased expression of defence genes downstream of the Imd, Toll, JNK and Jak-STAT pathways. We propose that either a Kay homodimer or a Jra/Kay heterodimer negatively regulates defence genes by repressing transcriptional activation of target genes in glia. In *VAPB^P58S^*, this negative regulation is weakened with age, allowing for a progressive increase in inflammation. The increase in inflammation is correlated with an age-dependent motor deficit. Glia inflammation, in turn, is predicted to lead to the secretion of inflammatory factors that affect glial:neuronal homeostasis and, ultimately, neuronal cell death.

In our model (Fig. 6), we propose that, in *Drosophila*, VAP regulates Toll, Jak-STAT JNK and Imd signalling via its action on upstream CKs. In the *VAP^P58S^* mutant, as VAP is a tether, maintaining MCSs, the relationship between the ER and other organelles such as mitochondria, lysosomes, Golgi, peroxisomes and the plasma membrane is disturbed (Borgese et al., 2021a,b; Kamemura and Chihara, 2019; Kodama et al., 2025; Kors et al., 2022; Voeltz et al., 2024), along with ER structure, function and dynamics being affected, leading to CK modulation. Multiple VAP interactors, as exemplified by STING, may also directly influence CK function. Specifically, we suggest that Tak1 function decreases with age in *VAP^P58S^* animals. This leads to reduced basal phosphorylation of Kay/Jra, which, in turn, de-represses defence genes (Fig. 6). The low-grade inflammation seen with increasing age would be a combined result of Tak1 modulation of its two arms – the ‘activating’ Rel arm versus the ‘negative-regulating’ Jra/Kay arm. The seat of the basal increase in inflammation is the glia, and sustained chronic low-grade inflammation leads to non-cell-autonomous spread to neurons, which in turn leads to neuronal degeneration.

The discovery of the VAPB:STING interaction (Ji et al., 2025) also highlights the possibility of STING being a significant mechanistic pathway for the initiation and progression of ALS (Almalki et al., 2025; Decout et al., 2021; Ji et al., 2025), with the source of the triggering DNA being damaged mitochondria in glial cells. This possibility, for the *VAP^P58S^* model, will be tested in future experiments in our laboratory. Another possible mechanism for increased inflammation in *VAP^P58S^* is related to the seminal discovery that the VAP^WT^-MSP domain can be cleaved and secreted from neurons (Tsuda et al., 2008). The extracellular MSP can then non-cell-autonomously signal to Eph receptors on neighbouring cells (Tsuda et al., 2008). Subsequently, in *Caenorhabditis elegans*, the secreted VAP-MSP domain has been shown to support neuromuscular homeostasis (Cottee et al., 2017; Han et al., 2012; Zein-Sabatto et al., 2021), with novel roles in regulating mitochondrial health and oocyte maturation, non-cell autonomously. Intriguingly, the *VAP^P58S^* mutant is resistant to cleavage and is unable to participate in cell:cell signalling. This raises the possibility that Eph or other target receptors on glial cells, in the absence of a regular MSP ‘anterograde’ signal from neurons, are prone to trigger inflammatory pathways. A recent review (Kamemura and Chihara, 2019) has discussed multiple possible non-cell-autonomous roles for VAP-MSP.

In summary, we find that Kay is a novel neuroinflammatory glial modulator in the *VAP^P58S^* model of ALS, suggesting an equivalent role for Fos in human microglia. Although the mechanism of this regulation or the link between VAP and Kay remains unclear, as is Kay's ability to regulate a broad set of inflammatory genes, further targeted mechanistic studies can open avenues for the development of new treatment strategies for ALS.

## MATERIALS AND METHODS

### Fly husbandry

All flies were reared at 25°C on standard cornmeal agar in a 12-h light-dark cycle. In specific cases, crosses were placed at 18°C to reduce developmental effects, then flies were collected on the first day of eclosion and transferred to vials at 25°C. All subsequent experiments were performed at 25°C. The fly lines used in this study are tabulated in Table S1.

### Generating the *VAP^P58S^* mutant using CRISPR/Cas9 genome editing

We employed a CRISPR/Cas9 single-stranded oligodeoxynucleotide (ssODN)-based strategy to generate a mutation at the *VAP* locus. In this strategy, a double-strand break (DSB) at the target locus is created by a guide RNA (gRNA), followed by repair of the DSB using a ssODN template containing the mutation. To generate this DSB at the *VAP* locus, as a first step, we designed and cloned a *VAP* gRNA containing the sequence 5′-TACGCAGTAGCGTTTCG-3′ (Fig. S1A) in the pBFv-U6.2B vector (Kondo and Ueda, 2013). The gRNA sequence targeted 3 bp upstream of the planned mutation site. The *pBFv-U6.2B VAP-gRNA* plasmid was injected into the *v^−^;attP40* line, and transgenics were scored by screening for *v^+^*. The stable balanced homozygous *VAP-gRNA* line was then crossed to a *v^−^*; *nanos-Cas9* (*nos-Cas9*) line (Kondo and Ueda, 2013) (Fig. S1B). Young embryos (0-2 h) of the F1 generation were collected. Approximately 700 embryos were injected with a synthesized ssODN (5′-TTGATCGAAAGGGAATTATCTTGCCGATGTTGCTACGTACGCAGTAGCGTTTCGGCGCGGTTGTCTTGATCTTGAAGACCAGAGGCAGAGCCGAGTTGTTG-3′) that contained the mutant sequence (Fig. S1D). Once the embryos developed into adult flies, straight-winged flies were crossed to balancers to preserve the genetic mutation and then stabilized, before screening for the mutation by genomic PCR. The ssODNA design incorporated a restriction digestion site 5′-TACGTA-3′, digested by SnaBI (Fig. S1C). Of the 359 lines stabilized, the incorporation of the template appeared efficient, as eight of the first set of 50 lines screened showed the presence of a SnaBI site (Fig. S1C). Four *VAP^P58S^* and three *VAP^WT^* lines, validated by sequencing, were maintained, while all remaining lines were discarded. These lines did not contain the gRNA (*v^−^*, *VAP^P58S^*) and were cleaned by crossing to a *v^−^* mutant line and rebalancing with FM7a.

### Immunohistochemistry

Adult brain immunohistochemistry to visualize vacuoles was done based on previously described protocols (Behnke et al., 2021; Bisht et al., 2026). Briefly, age-matched adult males of appropriate genotypes were anesthetized on ice, and brains were dissected in ice-cold 1× PBS. Brains were fixed in 4% formaldehyde (A805816, Polysciences, Inc.) in 0.5% PBT (0.5% Triton X-100 in 1× PBS) for 20 min. After four PBT washes of 15 min each, the tissues were incubated with Hoechst (1:1000, 10 mg/ml, Invitrogen) and Phalloidin-Alexa Fluor™ 488 (1:100, A12379, Thermo Fisher Scientific) for two nights at 4°C. Brains were washed five times in 1× PBT for 15 min each and once briefly in 1× PBS. Samples were mounted in ProLong™ Gold Antifade Mountant (P10144, Thermo Fisher Scientific) and imaged on Olympus Evident FV4000 and Leica SP8 confocal microscopes.

For antibody staining, brains were fixed in 4% formaldehyde in 0.5% PBT, blocked with 2% bovine serum albumin in 0.3% PBT, and incubated with primary antibodies anti-BiP (1:200, 3177, Cell Signaling Technology) or anti-VAP (1:500 in-house, rabbit polyclonal) overnight at 4°C. Secondary antibody goat anti-rabbit-Alexa Fluor™568 (1:1000, A11011, Thermo Fisher Scientific) was added for overnight incubation at 4°C. Hoechst was added in the penultimate wash before mounting. Images were acquired on a Leica SP8 confocal microscope. Immunohistochemistry for aggregate visualization was done as described in Thulasidharan et al. (2024).

### Image analysis

For vacuolar analyses, images were imported to Fiji (Schindelin et al., 2012), with the central brain analysed for vacuoles. Any area that did not show either nuclear or F-actin signal was considered a vacuole. A region of interest (ROI) was drawn around the vacuoles in the *z*-section in which they were largest. The number, average area per brain and area distribution of vacuoles were plotted using GraphPad Prism 10 (www.graphpad.com; RRID:SCR_002798).

For BiP puncta quantification, images were imported to Fiji, and six ROIs were selected per brain along the sub-oesophageal zone. Images were smoothened and manually thresholded, and puncta were counted using the 3D Objects Counter plugin. This count was divided by the volume of the ROI and plotted as a data point. Analysis for aggregate visualization was as described in Thulasidharan et al. (2024).

### RNA isolation, 3′ mRNA library preparation and sequencing

Total RNA was isolated from either 50 heads or 50 guts using trizol-chloroform extraction. For each sample, 3′ mRNA libraries were generated from four biological replicates using a QuantSeq 3′ mRNA-Seq Library Prep Kit FWD (015.96 and 191.91, Lexogen). Library concentrations were quantified with a Qubit™ dsDNA HS Assay Kit (Q32851, Thermo Fisher Scientific). Quality assessment and library size estimation was done using an HS DNA kit (5067-4626, Agilent) in an Agilent Bioanalyzer 2100 for individual libraries. Equimolar amounts of each library were pooled, and single-end 75 bp reads were sequenced on the Illumina NovaSeq X Plus platform.

### Read mapping, count generation, differential expression analysis and data visualization

Sequencing reads were aligned to the *D. melanogaster* reference genome (version dm6) using the STAR aligner. Gene-level expression quantification was carried out using HTSeq-count, which generated raw read counts for each gene based on the corresponding annotation. Differential gene expression analysis was performed using DESeq2 through pairwise comparisons of the biological conditions (Dataset 1). All computational steps, including alignment, quantification and statistical analysis, were conducted using the Bluebee platform and Kangooroo NGS data analysis tool. Matplotlib (version 3.10.0) and seaborn (version 0.13.2) libraries were used to plot heatmaps. VolcaNoseR was used for plotting volcano plots. Downstream analysis was done using DAVID and G-profiler. GO analysis was done using ShinyGo (version 0.77).

### qRT-PCR

RNA was extracted from 40-50 heads per genotype using trizol-chloroform extraction. 1 μg RNA was used to prepare cDNA using a cDNA reverse transcriptase kit (2911843, Thermo Fisher Scientific). For qRT-PCR, gene-specific primers were used, along with iTaq UniversalSyBr Green Supermix (1725124, Bio-Rad), in a qTOWER^3^ Series Real-Time PCR Thermal Cycler (Analytik Jena). Normalization was done using *rp49* as the housekeeping gene. Forward and reverse primers used for the experiment are provided in Table S2.

### Cloning and construct generation for *kay/kay^SCR^* OE lines

cDNA for *kay* was amplified from the *Drosophila* gold collection library using specific primers. *kay^K357R^* was generated using site-specific mutagenesis to make a SUMO conjugation-resistant form of Kay (*kay^K357R^/kay^SCR^*) by mutating lysine to arginine. The second-chromosome UAS transgenics for *kay^WT^* and *kay^K357R^* were generated by targeted insertion at attP40 using the pUASp-attB vector obtained from the *Drosophila* Genomics Resource Centre (1358). The transgenics were screened, balanced and then used for experiments. Primers used for the experiment are listed in Table S2. In addition to *UAS*-*kay^WT^* and *UAS*-*kay^K357^*, the BDSC:7213 (*UAS-kay^WT^*) line from BDSC was used to validate and extend our results. Expression of all three lines was pupal lethal using constitutive Gal4 drivers, as is expected with *kay* OE. Fold changes in levels of expression for the lines were measured by qRT-PCR as 17-, 12- and 10-fold, for *UAS*-*kay^WT^*, *UAS*-*kay^K357^* and *BDSC UAS-kay*, respectively.

### Motor assay

Motor performance was assessed using the startle-induced negative geotaxis climbing assay. For each replicate, 30 F1 males and females were collected for each genotype and transferred to vials. Each vial contained 15 or fewer age-matched flies. The motor assay was performed for each genotype on days 5, 10, 15, 20 and 25. All the experiments were performed in triplicate. Each set of 30 flies was transferred to a 250 ml borosilicate cylinder, and the flies were allowed to acclimatize for 1 min. The cylinder was then tapped to make all the flies settle at the bottom of the cylinder, and the timer was started. The flies were allowed to climb for 40 s, and the numbers of flies in each zone of the cylinder were counted at the end of 40 s. Flies that did not climb were scored 0 (non-climbers), flies that climbed to the 80 ml (7.5 cm) mark were scored 3 (poor climbers), and the flies that crossed the 80 ml mark were scored 5 (good climbers) for both males and females. The experiment was repeated three times for each set, with a 1-min resting period between repeats. The flies were then transferred back to the vials by anesthetizing with CO_2_. To ensure that CO_2_ did not affect the climbing performance, the flies were exposed to CO_2_ only after each day's trial. Flies were flipped every third day for males and every fourth day for females. The scores were used to calculate the climbing index (Azuma et al., 2014; Tendulkar et al., 2022). Statistical analysis was performed using two-way ANOVA, and Tukey's test was used to perform multiple comparisons.

### Lifespan and survival analysis

Survival assays were conducted at 25°C. For each replicate, 80-100 F1 male and female flies per genotype were collected and distributed into vials, with no more than 15 age-matched flies per vial. The flies were flipped every third day in case of males and every fourth day in case of females to avoid death by sticking to the media. Dead flies in each vial were counted daily until the last fly of every genotype was dead. All experiments were performed in triplicate. The data were analysed using the log-rank test. The ML for each genotype was recorded.

### Statistical analysis

Experiments were performed with at least three biological replicates (*N*=3). For all QuantSeq experiments, *N*=4. The *n* (number of samples), *N* values and statistical tests used are listed in the figure legends for each experiment. GraphPad Prism 8.0 was used for data plotting and statistical analysis.

### Biosafety

The protocols/experiments used in our research were approved by the Indian Institute of Science Education and Research (IISER) Institutional Biosafety Committee (IBSC), which, in turn, is monitored by the Review Committee on Genetic Manipulation (RCGM), Department of Biotechnology, Government of India.

### AI usage

During the preparation of this work, the authors used Grammarly to correct grammatical and sentence-structure errors. The authors take full responsibility for the content of the published article.

## Supplementary Material

10.1242/dmm.052810_sup1 Supplementary information

Dataset 1. Pairwise comparisons of differentially expressed genes for transcriptomic datasets.
