# Supplementary material for: Fos regulates age-dependent neuroinflammation in a VAPP58S model of amyotrophic lateral sclerosis
Source: Dis Model Mech. 2026 Jul 24;19(7):dmm052810. doi: 10.1242/dmm.052810 (PMC13446562; doi:10.1242/dmm.052810)
Supplement: Supplementary file 1 [file dmm-19-052810-s1.pdf]

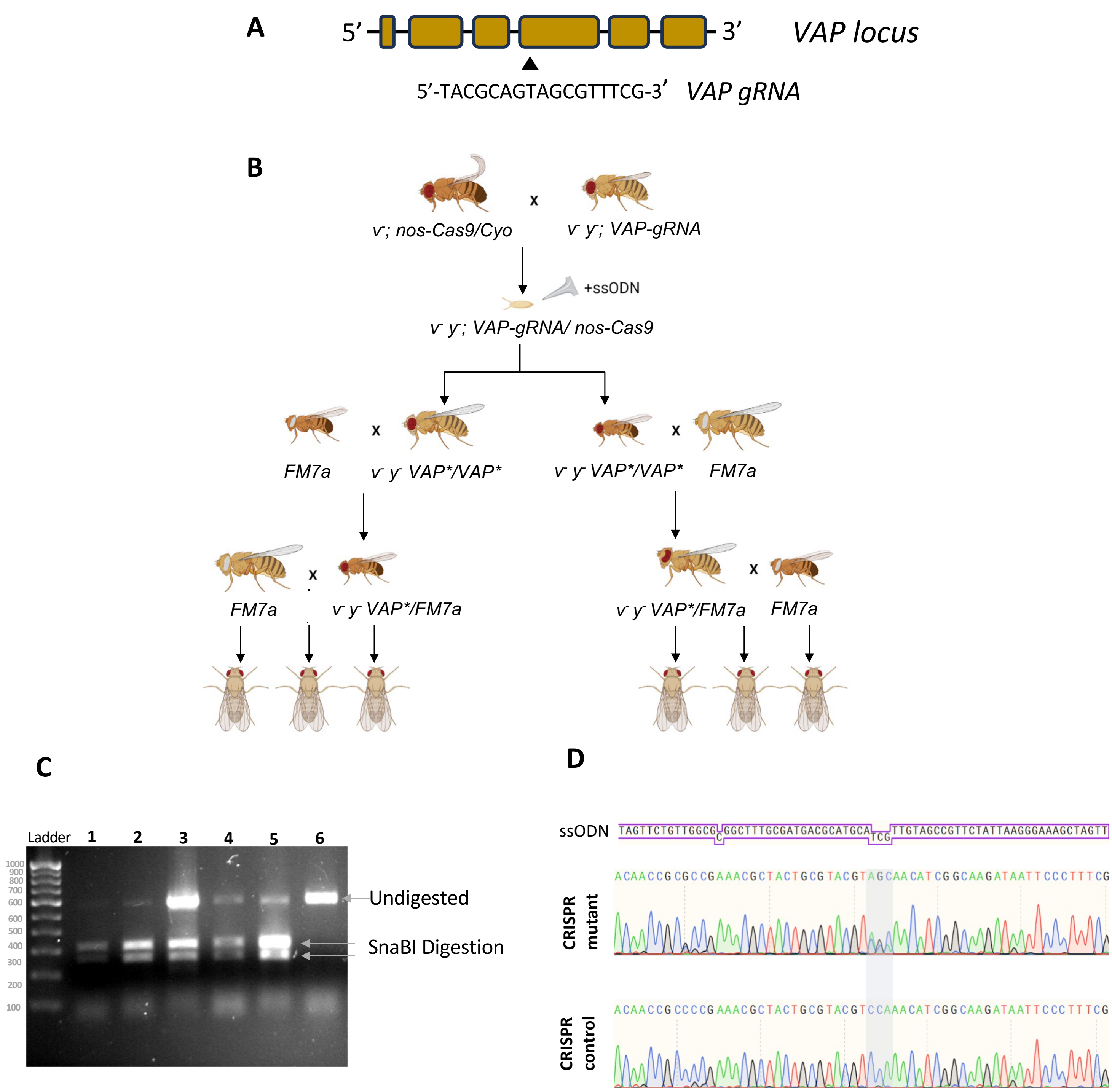

Fig. S1. Generation of *VAP*^{P58S} mutant fly lines using a CRISPR-Cas9 strategy

A. Schematic for *VAP33A* genetic locus and the sequence of the *gRNA* designed to target the 4th exon of *VAP*.

B. A *VAP* guide-RNA transgenic line was generated (see Materials & Methods), balanced, and crossed to *nos-Cas9.* 700 F1 embryos were injected and 359 straight-wing flies were further balanced, expanded and stabilized.

C. 50 (out of 359) lines were initially screened using genomic PCR and *SnaBI* digestion. 8 lines showed digestion (e.g., Lines 2,4,5 indicating the replacement of part of the *VAP* genomic region with the mutant *ssODNA* sequence.

D. Eight lines were sequenced, with seven showing the replacement of codon CCA with AGC and no other mutations in the *VAP* genomic region. These were defined as 'validated' *VAP*^{P58S} lines and were characterized for lifespan and startle assays. Three lines that did not show *SnaBI* digestion (panel C, lane 3, 6) were also sequenced and maintained as *VAP*^{WT} (CRISPR Control) lines.

Disease Models & Mechanisms • Supplementary information

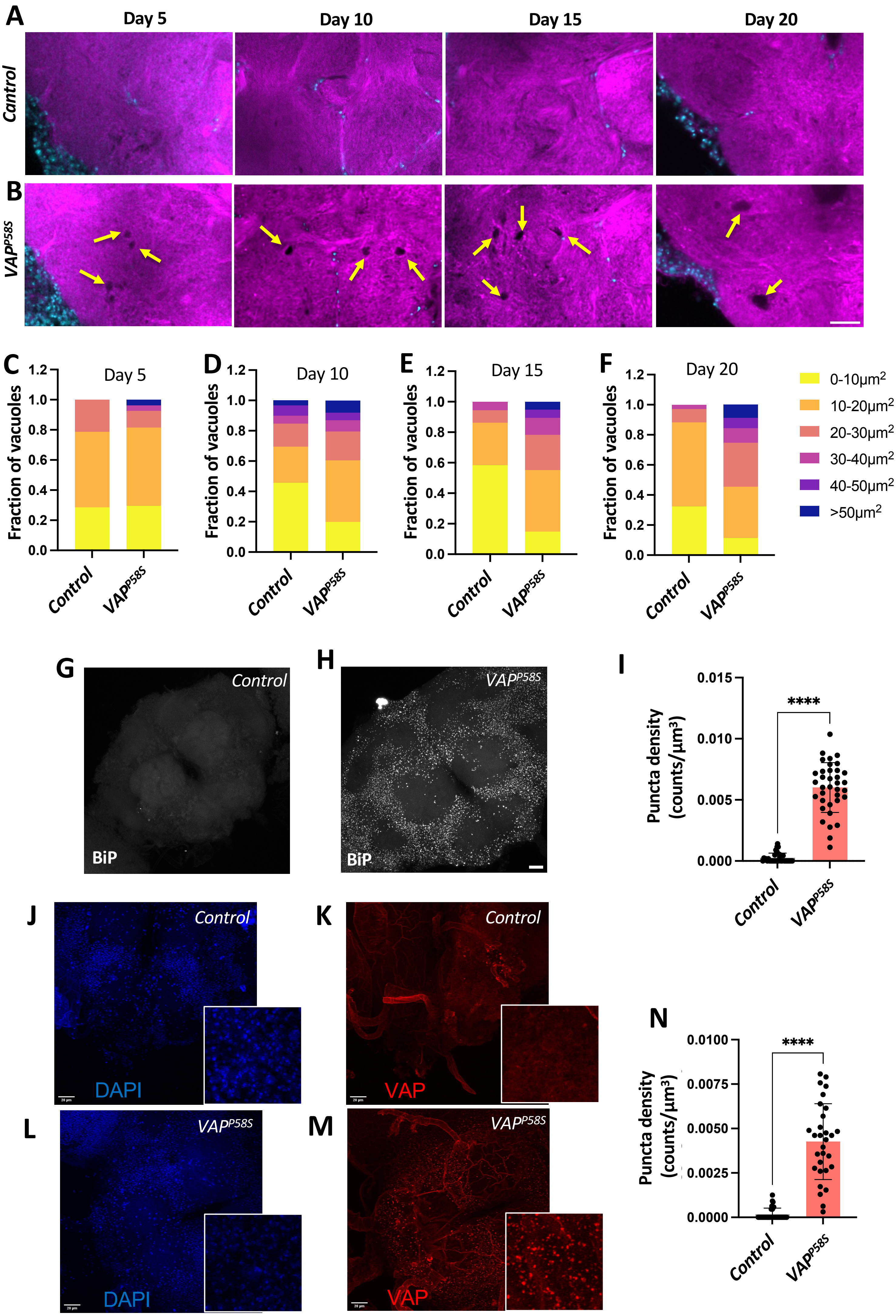

Disease Models & Mechanisms • Supplementary information

Fig. S2. ***VAP^{P58S}*** **mutant shows markers of frank neurodegeneration:**

A-B. Single section of confocal images showing nuclear stain Hoechst (cyan) and F-actin stain Phalloidin (magenta). (A) *Canton S*, (B) *VAP^{P58S}*. Arrows point to pathological vacuoles.

C-F. Stacked bar plot showing a histogram of vacuole area categorized as indicated in figure legend. (C) 5-day-old flies, (D) 10-day-old flies, (E) 15-day-old flies, (F) 20-day-old flies. At least 5 brains of each genotype were used for quantification.

G-H. MIP of confocal images showing BiP in 21-day-old flies of genotypes (G) *Canton S* and (H) *VAP^{P58S}*. Scale bar represents 20um.

I. Scatter plot with bar shows quantification of BiP puncta density. Genotypes are *Canton S* (n=6, 0.0002±0.0004) and *VAP^{P85S}* (n=6, 0.0060±0.0020), p<0.0001. Statistical significance was calculated using Mann-Whitney test. Bar and error bars denote mean and SD, ****p<0.0001.

J-M. MIP of confocal images showing nuclear stain DAPI (blue) and VAP (red) in 15-day-old flies. (J-K) shows control genotype (L-M) shows *VAP^{P58S}*. Insets are magnified images. All scale bars represent 20µm.

N. Scatter plot with bar shows quantification of VAP puncta density. Genotypes are *control* (n=5, 0.0002±0.0003) and *VAP^{P85S}* (n=5, 0.0043±0.0021), p<0.0001. Statistical significance was calculated using Mann-Whitney test. Bar and error bars denote mean and SD, ****p<0.0001.

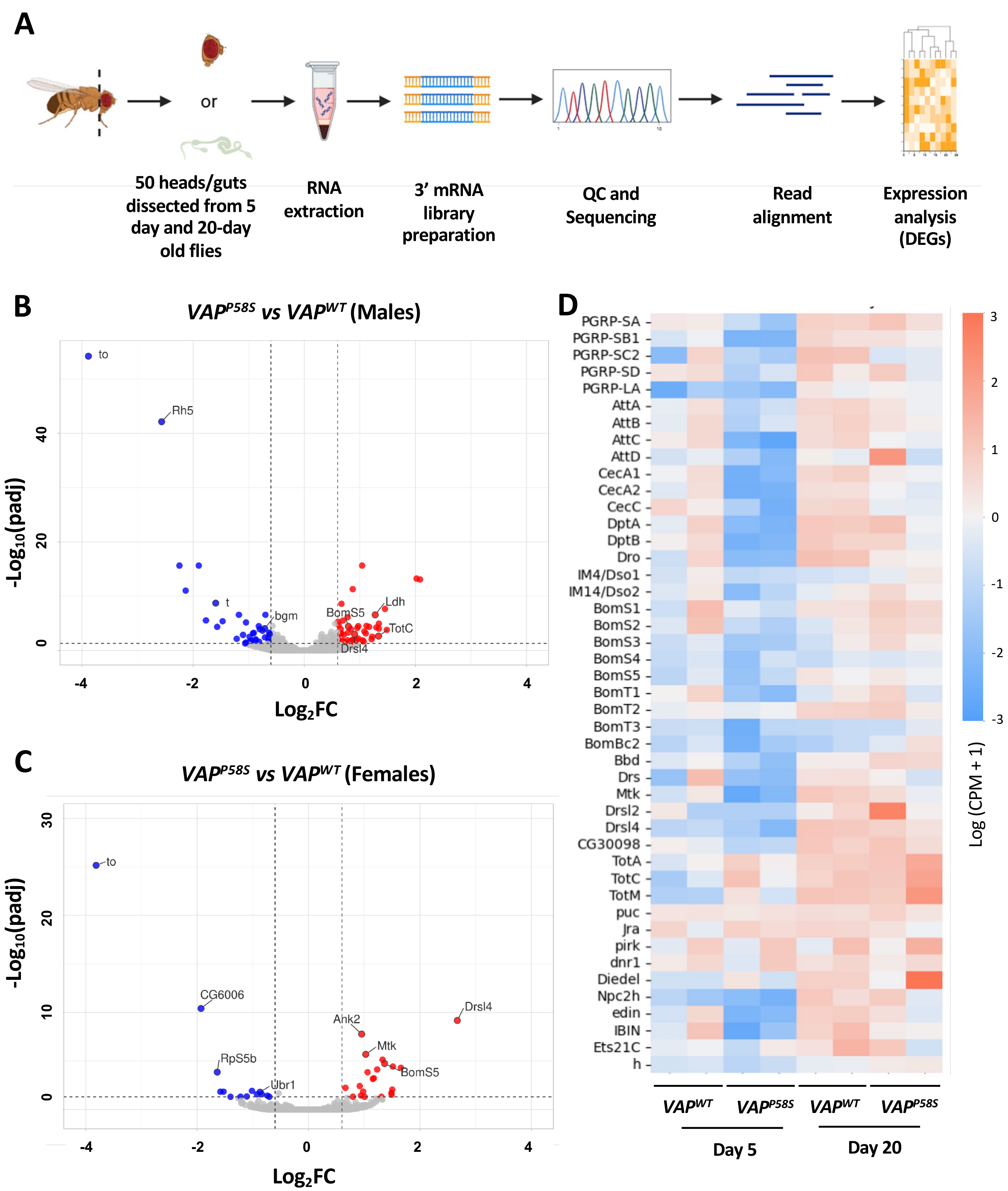

Fig. S3. Male and female head transcriptomics for *VAP^{P58S}* and *VAP^{WT}*

A. Diagrammatic representation of the experimental workflow for 3'mRNA sequencing. 50 heads or guts per genotype per timepoint were crushed in trizol. RNA was extracted for 3'mRNA library preparation. The completed libraries were sent for quality control (QC) and sequencing following which reads were aligned and the downstream analysis to obtain DEGs was performed

B-C. Volcano plots for males and females respectively for the complete dataset for *VAP^{P58S}* and *VAP^{WT}*, highlighting a few immune genes and some common genes in the two sexes

D. Heatmap showing the normalized expression counts for immune genes significantly differentially expressed between *VAP^{P58S}* and *VAP^{WT}* at day 5 & 20.

A

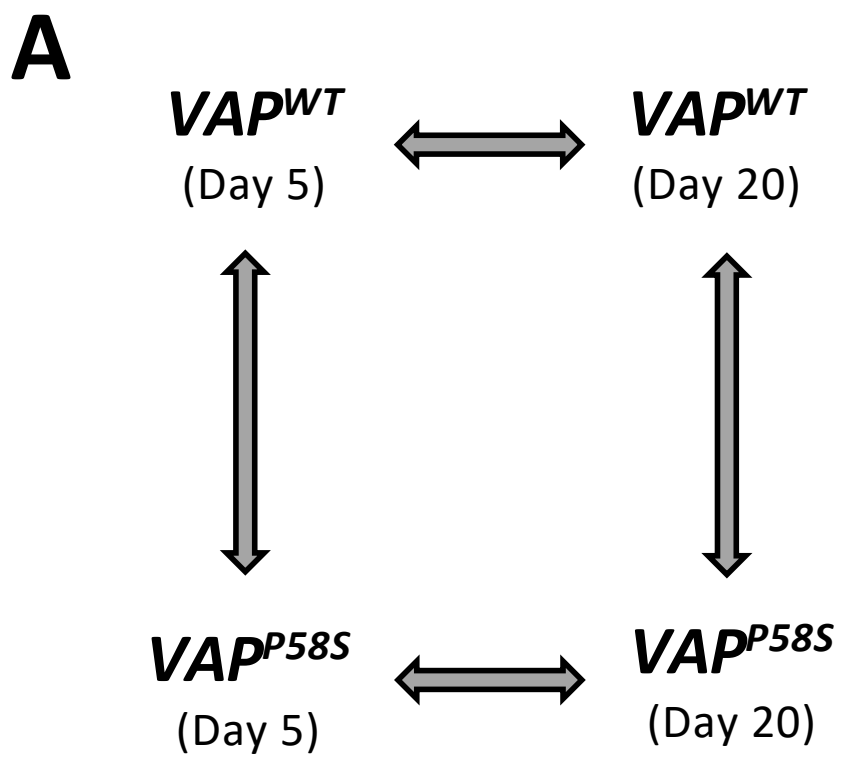

B

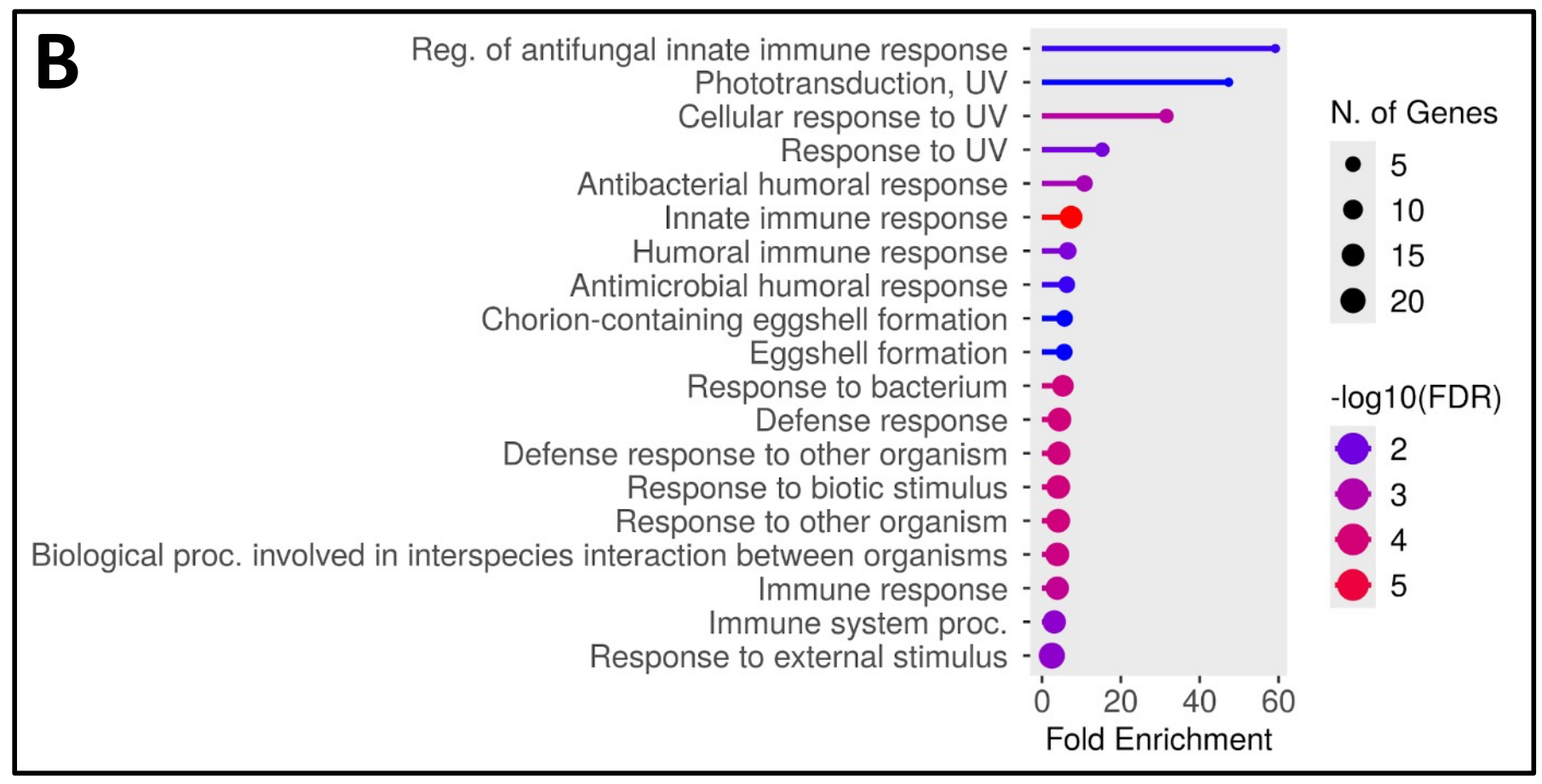

C

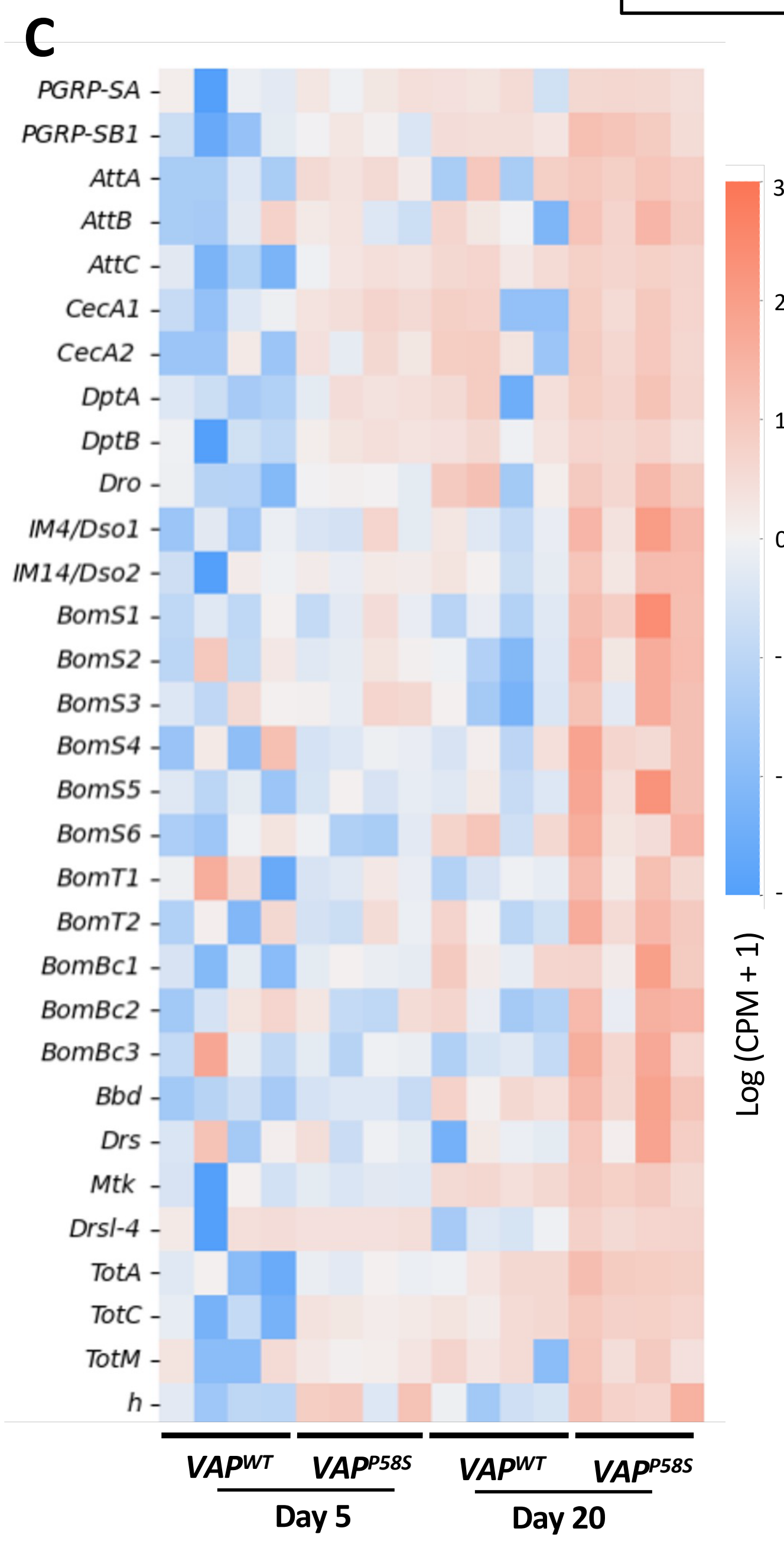

D

			Ageing		Disease	
			20d vs 5d		5d	20d
	Flybase ID	Flybase Symbol	VAP^{WT}	VAP^{P58S}	VAP^{P58S} vs VAP^{WT}	
Imd	FBgn0030310	*PGRP-SA*	0.81		0.80	0.83
Imd	FBgn0043578	*PGRP-SB1*	1.89	1.93	1.19	1.79
Imd	FBgn0012042	*AttA*	1.03			1.75
Imd	FBgn0041581	*AttB*		1.23		1.39
Imd	FBgn0041579	*AttC*	3.47	1.59	2.48	1.91
Imd	FBgn0000276	*CecA1*	1.21	1.15	2.34	1.13
Imd	FBgn0000277	*CecA2*	1.31	1.60		1.60
Imd	FBgn0004240	*DptA*	2.11	1.13	2.12	1.23
Imd	FBgn0034407	*DptB*	2.16	1.65	2.04	1.42
Toll	FBgn0010388	*Dro*	2.03	1.55	1.01	1.80
Toll	FBgn0040653	*IM4/Dso1*		0.80		0.93
Toll	FBgn0067905	*IM14/Dso2*		0.86		0.97
Toll	FBgn0034329	*BomS1*		1.01		1.00
Toll	FBgn0025583	*BomS2*		0.79		0.83
Toll	FBgn0040736	*BomS3*				
Toll	FBgn0034330	*BomS4*		0.62		0.64
Toll	FBgn0040734	*BomS5*		1.21		1.24
Toll	FBgn0040733	*BomS6*	0.70	0.95		1.14
Toll	FBgn0262838	*BomT1*	-0.95	1.17	-0.81	1.16
Toll	FBgn0040735	*BomT2*		1.07		1.06
Toll	FBgn0034328	*BomBc1*	1.14	0.89		1.35
Toll	FBgn0034331	*BomBc2*		0.70		0.68
Toll	FBgn0040582	*BomBc3*	-0.77	1.38		1.26
Toll	FBgn0034512	*Bbd*	0.98	1.21		1.26
Toll	FBgn0283461	*Drs*	-0.76	0.78		1.03
Toll	FBgn0014865	*Mtk*	2.06	3.06		2.86
Toll	FBgn0052282	*Drsl-4*	-1.46	1.24		1.30
Jak/STAT	FBgn0028396	*TotA*	1.85	2.46	0.72	
Jak/STAT	FBgn0044812	*TotC*	2.73		2.08	2.05
Jak/STAT	FBgn0031701	*TotM*		1.37		1.27
TF	FBgn0001168	*h*			1.20	

-1.5 to -2	-1 to -1.5	-0.5 to -1	0.5 to 1	1 to 1.5	1.5 to 2	2-2.5	2.5-3	3-3.5

E

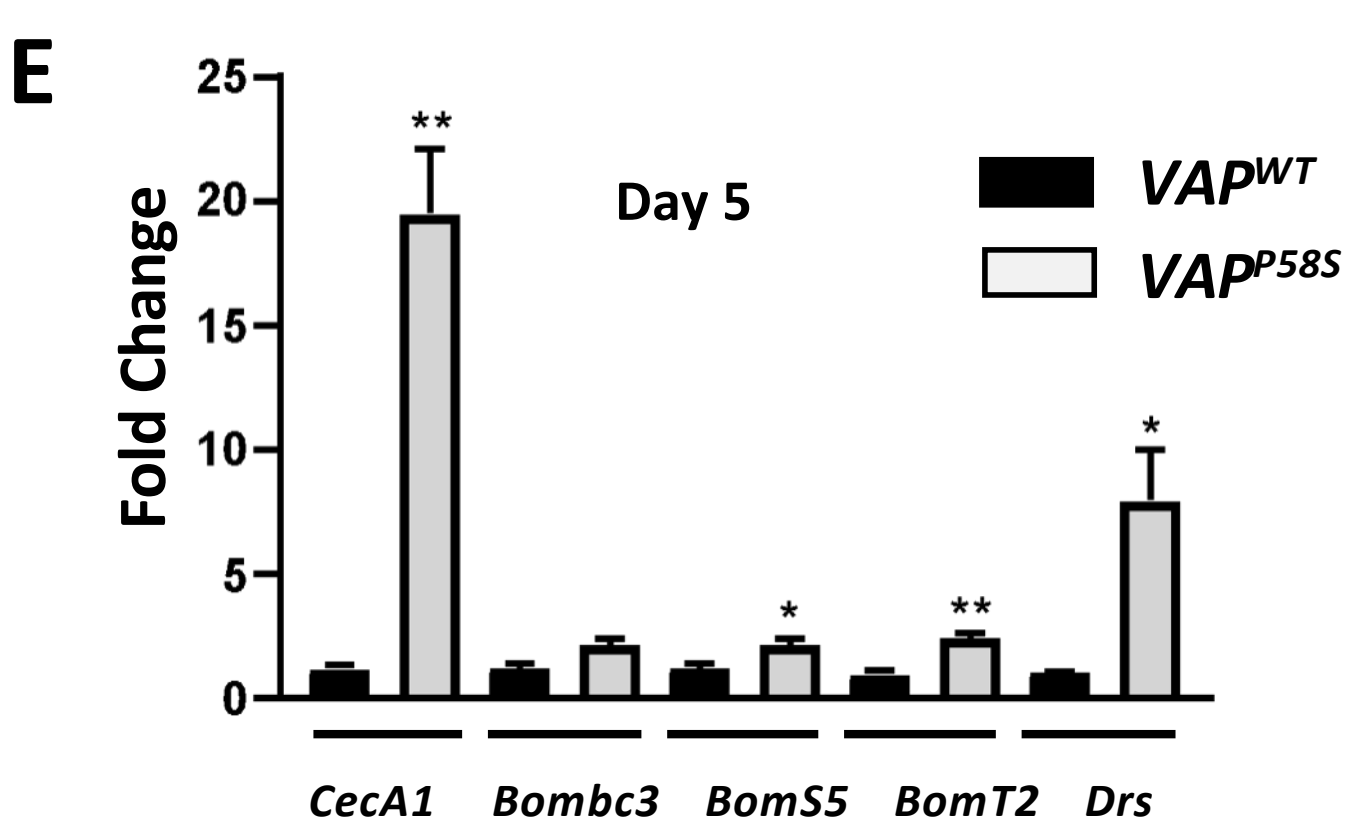

F

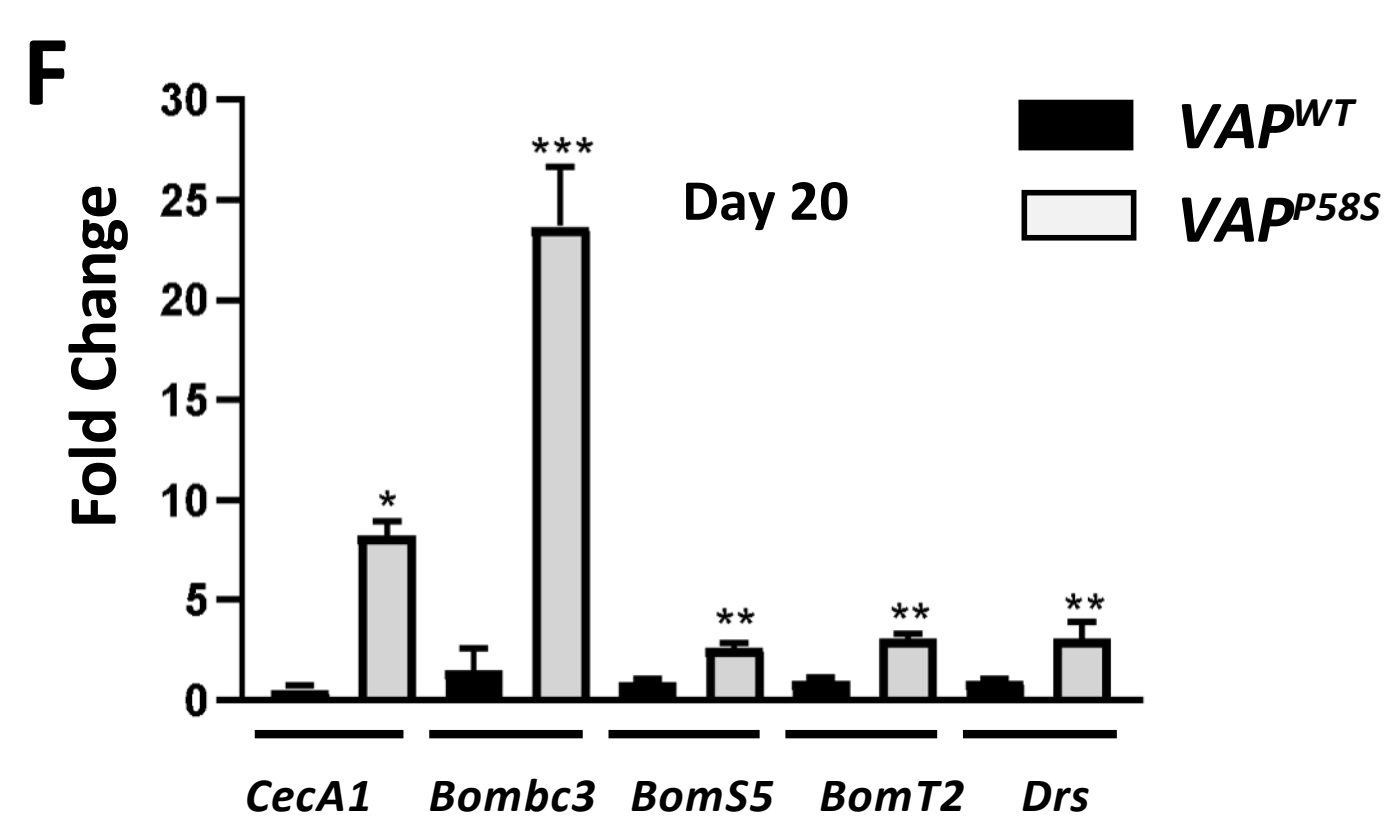

Disease Models & Mechanisms • Supplementary information

Fig. S4. Transcriptomics on *VAP*^{P58S} female heads showing enhanced age-dependent neuroinflammation

A. Genotypes and timepoints used for the 3' mRNA sequencing and the pairwise comparison done to obtain Differentially Expressed Genes (DEGs).

B. Gene Ontology (GO) enrichment analysis for significantly differentially expressed genes between *VAP*^{P58S} and *VAP*^{WT} at days 5 and 20 combined

C. Normalized expression counts for immune genes significantly differentially expressed between *VAP*^{P58S} and *VAP*^{WT} ,5d and 20d combined, depicted in the form of a heatmap

D. Summary table for immune gene transcripts categorized based on pathway/molecular function. Values represented as $\log_2$FC with the colour code given at the bottom.

E-F. qRT-PCR for the Imd pathway target *CecA1* and Toll pathway targets *Bombc3*, *BomS5, BomT2* and *Drs* at days 5 and 20 respectively for *VAP*^{WT} and *VAP*^{P58S} heads. The Y-axis shows $\log_2$FC values normalised to the housekeeping gene *rp49*. Values shown as mean + SEM. n= 40-50, N=3. Two-way ANOVA was used for statistical analysis. Tukey's test was used for multiple comparison. *$p< 0.05$, **$p< 0.01$, ***$p< 0.001$.

Disease Models & Mechanisms • Supplementary information

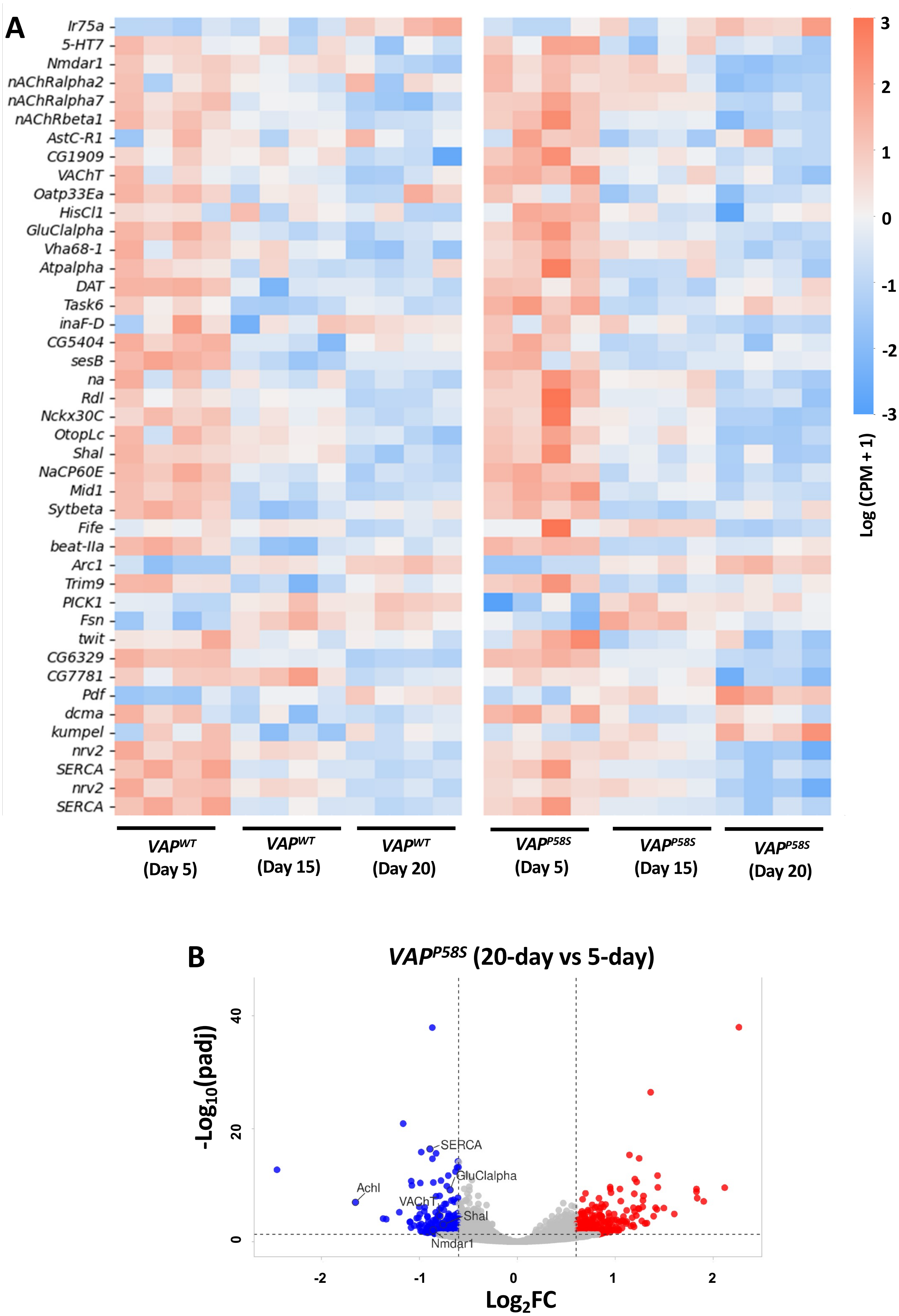

Fig. S5. Heatmap and Volcano plot for *VAP^{P58S}* male aging

A. Heatmap showing the normalized expression counts for synaptic genes across genotypes and timepoints
B. Volcano plots representing up and downregulated genes for the complete dataset for *VAP^{P58S}*, 20d vs 5d. A few downregulated synaptic genes are highlighted

Up regulated

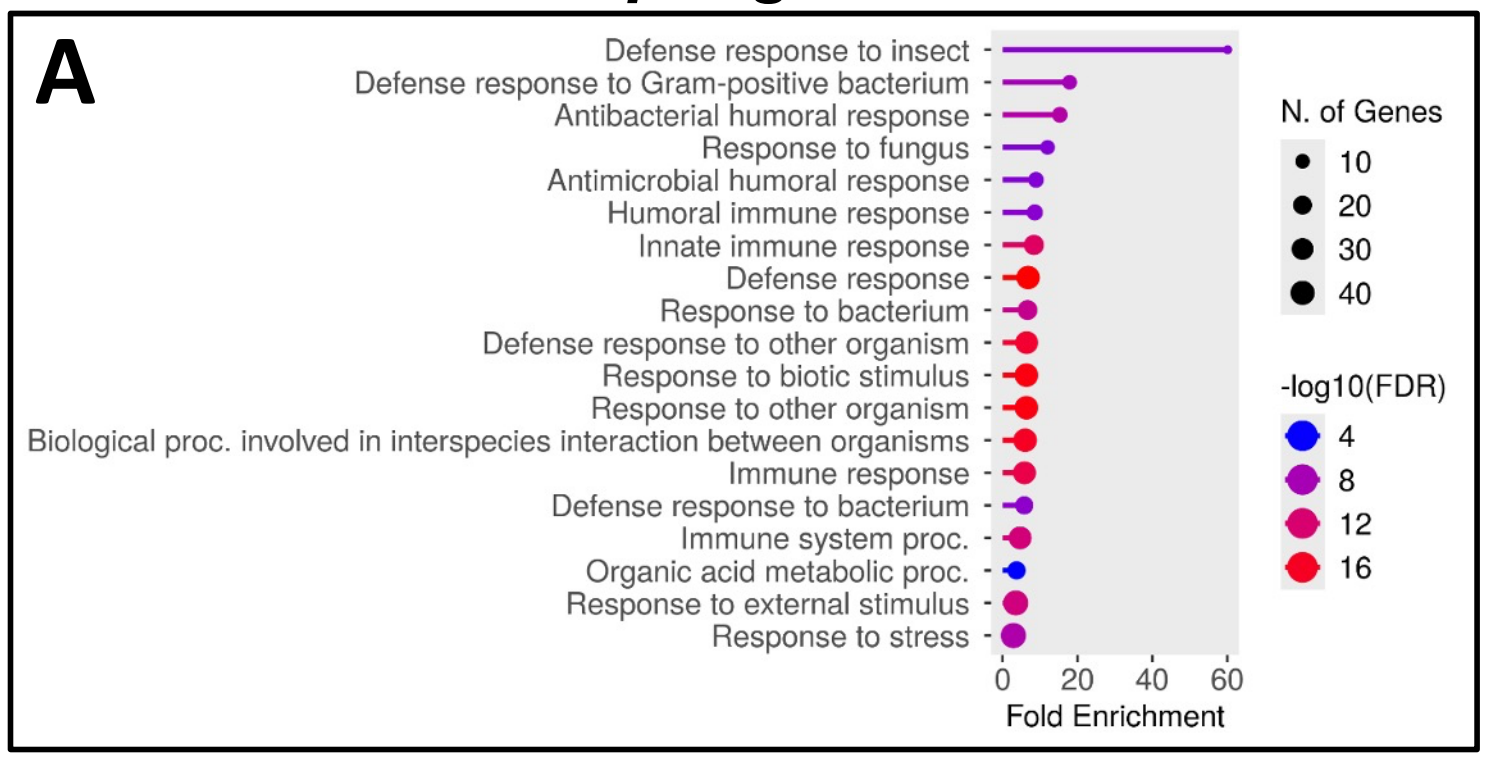

Down regulated

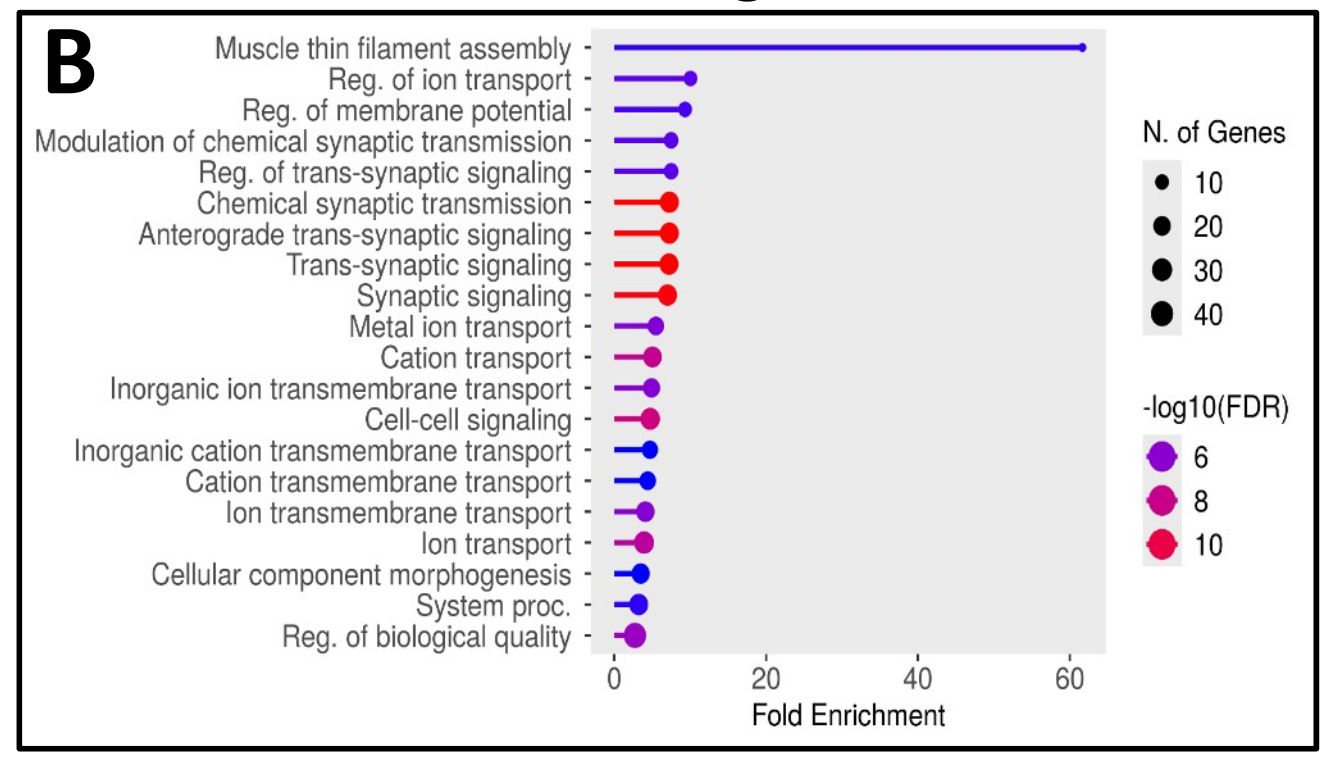

C

	Flybase ID	Flybase Symbol	VAP^{WT} 20d	VAP^{P58S} 20d
Neurotransmitter receptors	FBgn0024944	*Oamb*	0.98	
	FBgn0086778	*nAChRalpha7*		-1.01
	FBgn0000038	*nAChRbeta1*		-0.95
	FBgn0011582	*Dop1R1*	0.63	
Transporters and Ion channels	FBgn0033196	*HisT*		-0.63
	FBgn0024963	*GluClalpha*		-0.66
	FBgn0003380	*Sh*		-0.69
	FBgn0261794	*kcc*		-0.80
	FBgn0259994	*OtopLa*		-0.64
	FBgn0259145	*CG42260*		-0.66
	FBgn0004244	*Rdl*		-0.65
	FBgn0028704	*Nckx30C*		-0.64
	FBgn0039915	*Gat*	-0.63	-0.69
	FBgn0259150	*OtopLc*	0.68	
	FBgn0039081	*Irk2*	-0.70	-0.82
	FBgn0085434	*NaCP60E*		-0.82
Synaptic vesicle release machinery	FBgn0004242	*Syt1*		-0.67
	FBgn0033876	*Syngr*		-0.74
	FBgn0032901	*sky*		-0.62
Synapse formation and plasticity	FBgn0038498	*beat-IIa*		-0.96
	FBgn0033926	*Arc1*		0.74
	FBgn0038975	*Nrx-1*		-0.65
	FBgn0030174	*CG15312*	0.65	-0.72
	FBgn0040505	*Alk*		-0.63
	FBgn0267001	*Ten-a*		-0.67
Synaptic signalling and Modulators	FBgn0029663	*CG10804*		-0.71
	FBgn0036007	*path*		0.66
	FBgn0263006	*SERCA*	-1.11	-0.77
	FBgn0030897	*Frq1*	-0.61	-0.71

-1.5 to -2	-1 to -1.5	-0.5 to -1	0.5 to 1	1 to 1.5	1.5 to 2	2-2.5	2.5-3	3-3.5

Disease Models & Mechanisms • Supplementary information

D

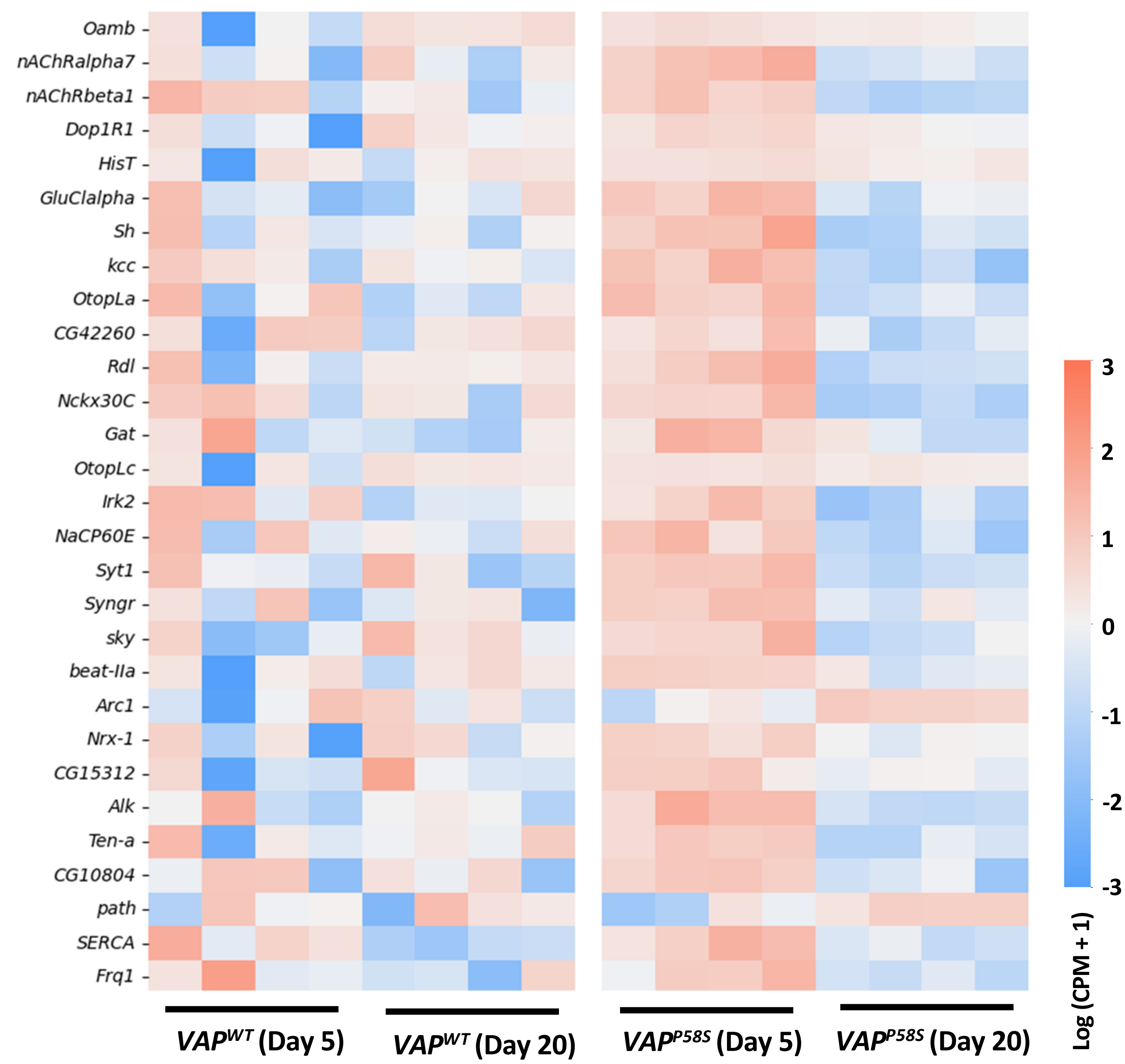

E

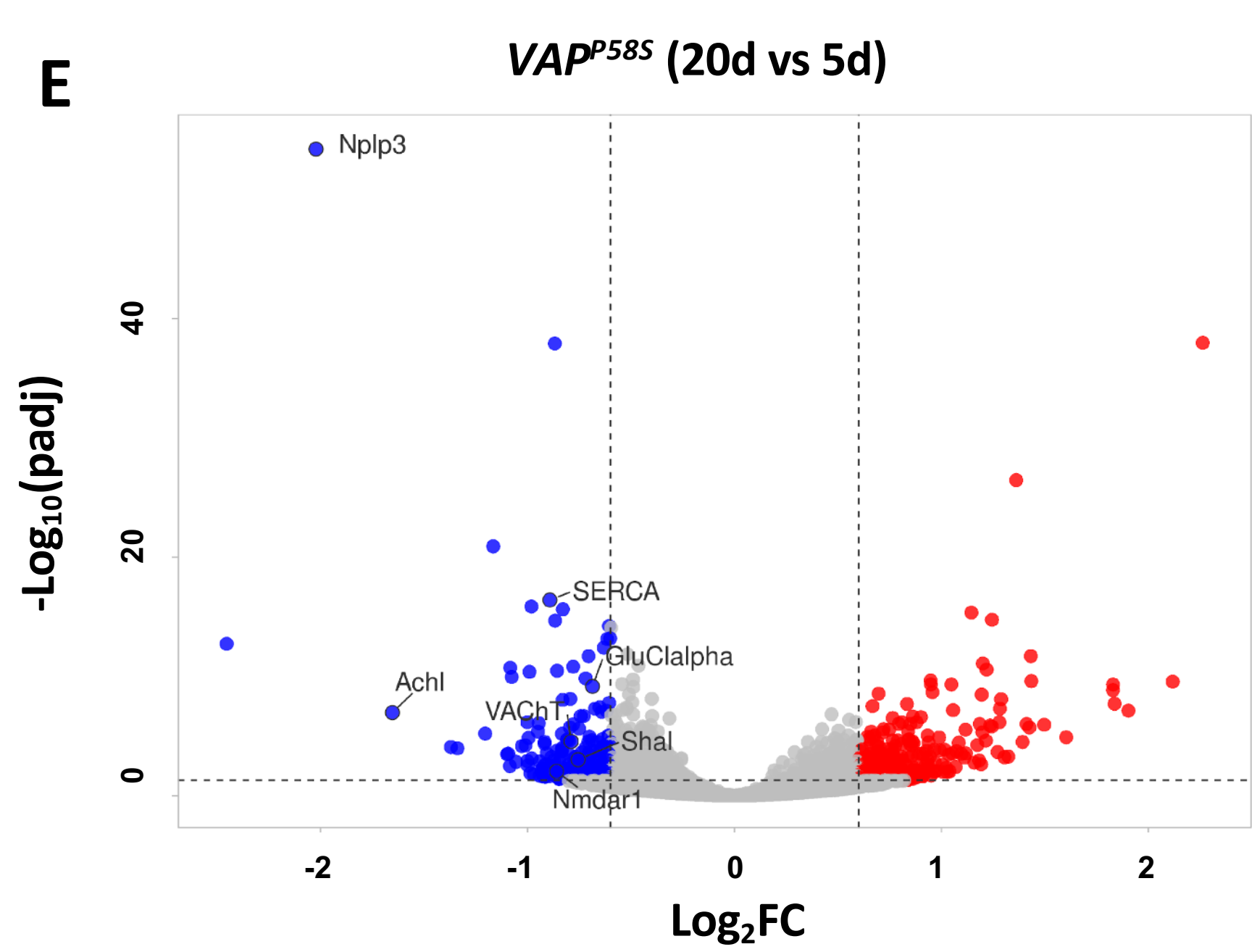

Disease Models & Mechanisms • Supplementary information

Fig. S6. Transcriptomic study in *VAP^{P58S}* females showing changes in synaptic signalling with age.

A. Gene Ontology (GO) enrichment analysis for significantly upregulated genes in *VAP^{P58S}* between days 5 and 20

B. Gene Ontology (GO) enrichment analysis for significantly downregulated genes in *VAP^{P58S}* between days 5 and 20

C. Tabular representation of log_2FC values for synaptic gene transcripts. The genes have been categorized into the functions they perform. The table shows comparison of *VAP^{P58S}* at day 20 with *VAP^{P58S}* at day 5 . The colour code is given at the bottom.

D. Heatmap showing the normalized expression counts for synaptic genes across genotypes and timepoints

E. Volcano plots representing up and downregulated genes for the complete dataset for *VAP^{P58S}*, 20d vs 5d. A few downregulated synaptic genes are highlighted

Disease Models & Mechanisms • Supplementary information

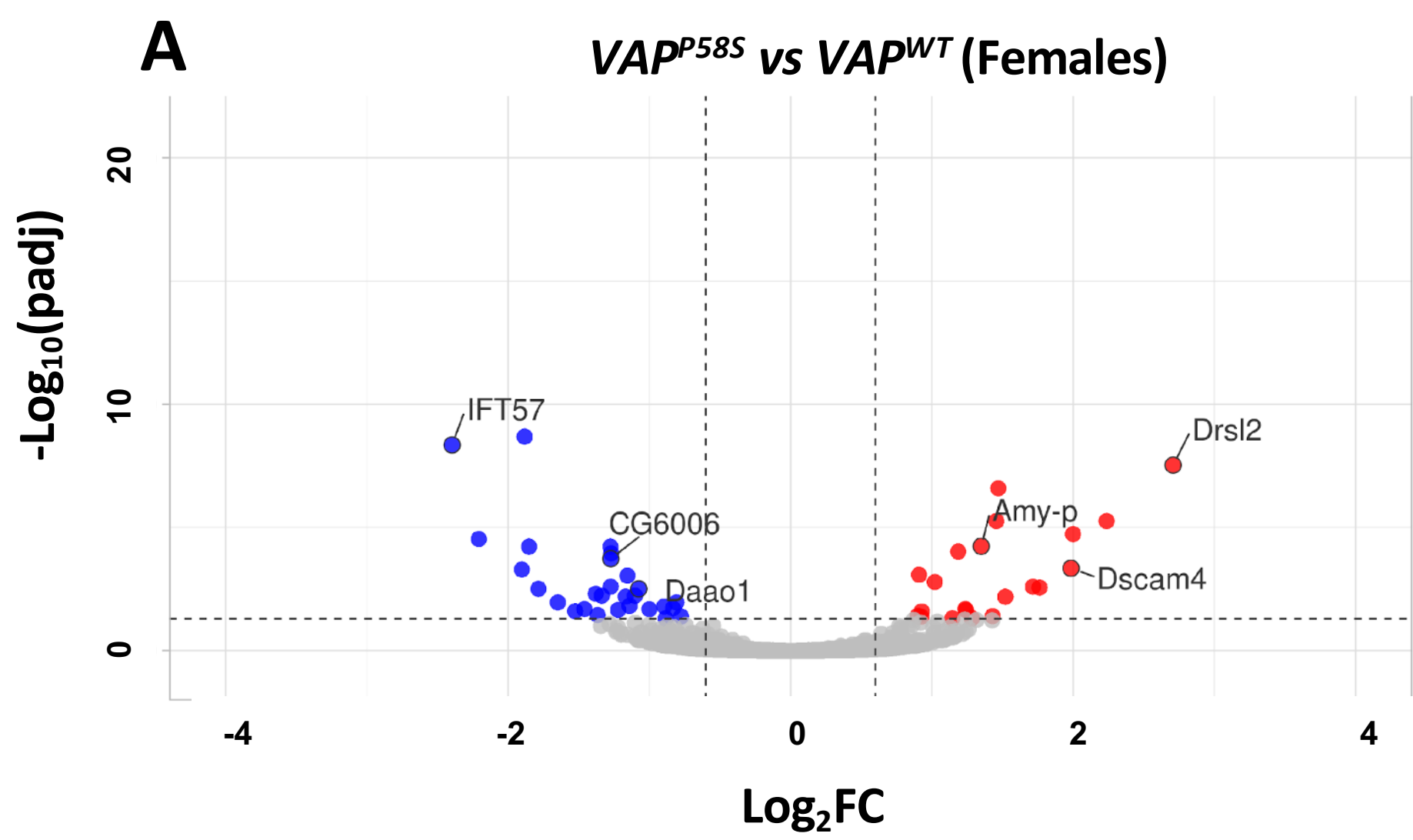

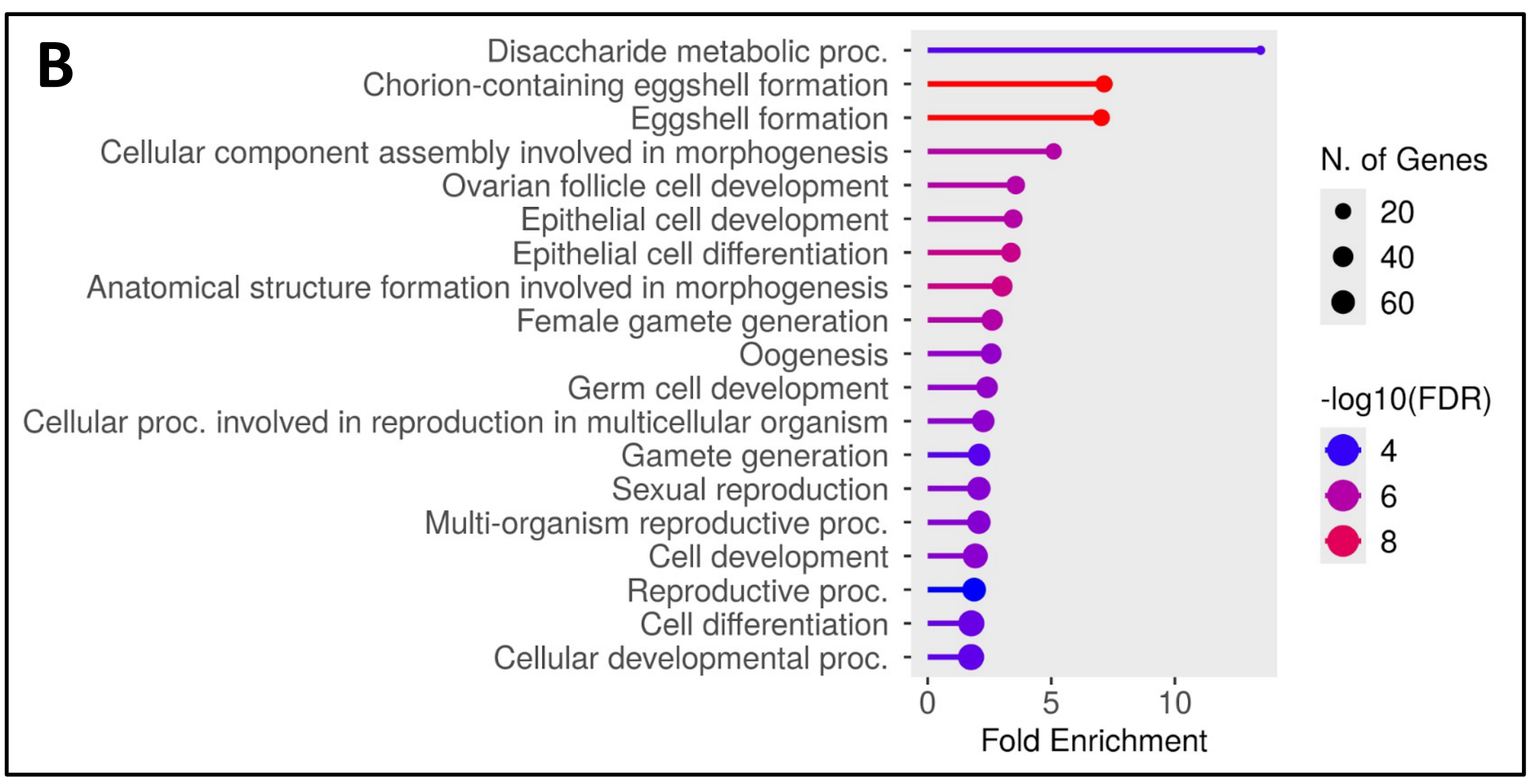

C

		Ageing		Disease	
		20d vs 5d		5d	20d
Flybase ID	Flybase Symbol	*VAP*^{WT}	*VAP*^{P58S}	*VAP*^{P58S} vs *VAP*^{WT}	
FBgn0043575	*PGRP-SC2*				0.62
FBgn0041579	*AttC*		2.06		
FBgn0052279	*Drsl2*		1.74		2.71
FBgn0028396	*TotA*		1.92		
FBgn0044812	*TotC*		1.47		
FBgn0265577	*IBIN*		1.80		
FBgn0001168	*h*			1.29	1.04

-1.5 to -2	-1 to -1.5	-0.5 to -1	0 to -0.5	0 to 0.5	0.5 to 1	1 to 1.5	1.5 to 2	2-2.5	2.5-3	3-3.5

Disease Models & Mechanisms • Supplementary information

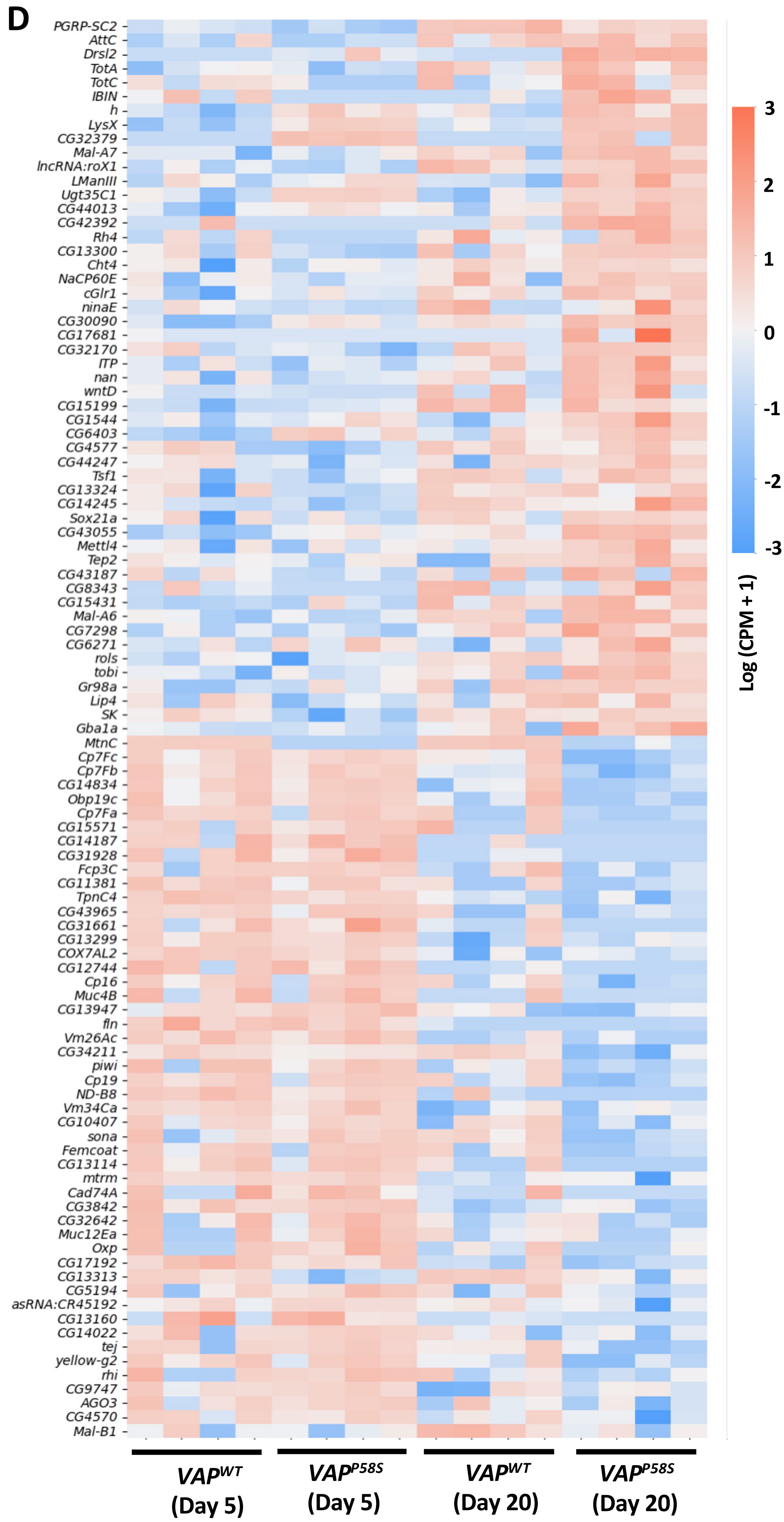

Disease Models & Mechanisms • Supplementary information

Fig. S7. Gut transcriptomics for *VAP*^{P58S} females

A. Volcano plots for the genes of the entire dataset with a few important genes being highlighted

B. Gene Ontology (GO) enrichment analysis for significantly differentially expressed genes between *VAP*^{P58S} and *VAP*^{WT}

C. Summary table for immune gene transcripts. Values represent $\log_2$FC with the colour code given at the bottom.

D. Subset of genes significantly differentially expressed between *VAP*^{P58S} and *VAP*^{WT} for days 5 and 20 combined, plotted as a heatmap showing normalized expression counts

Disease Models & Mechanisms • Supplementary information

Suppl. Figure 8

A

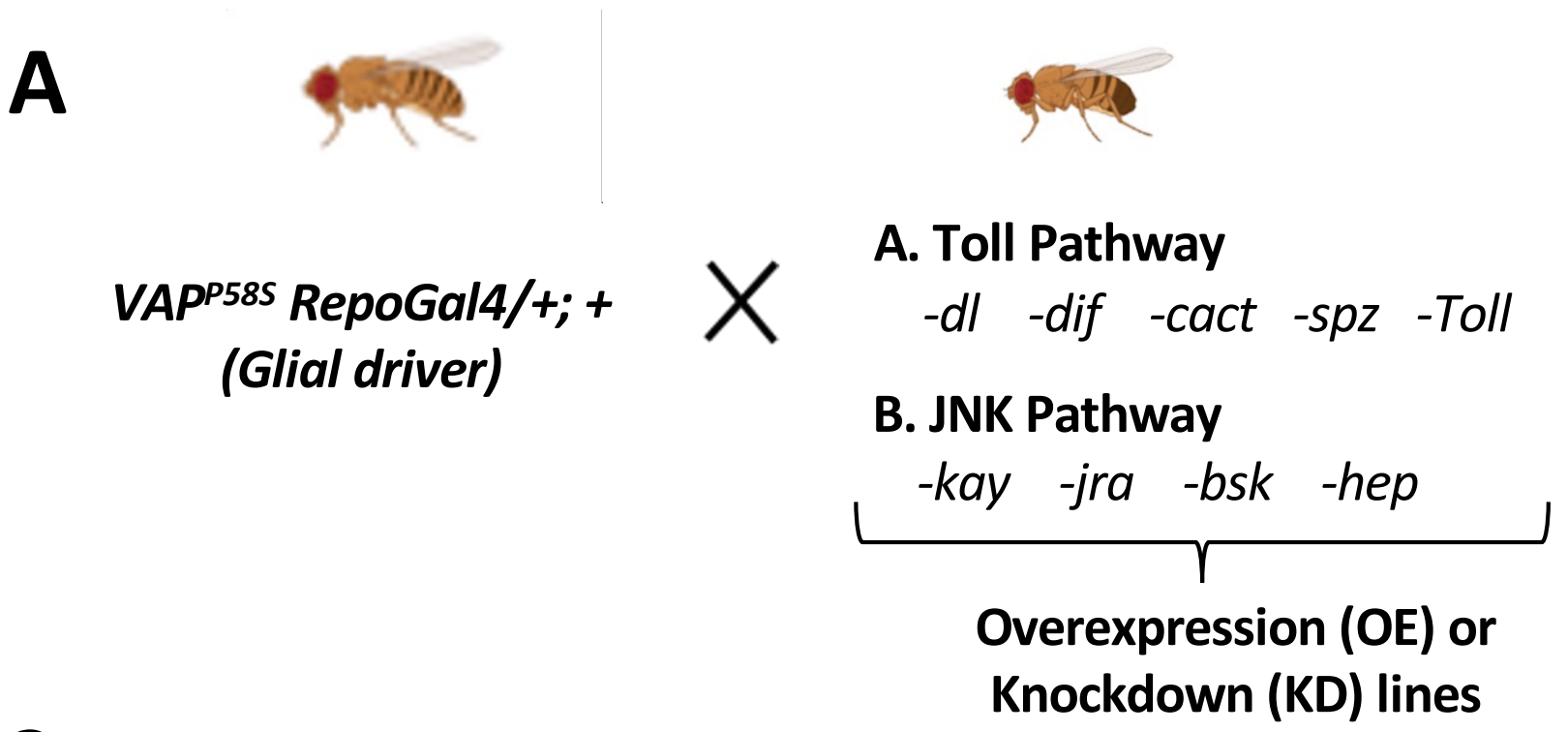

B

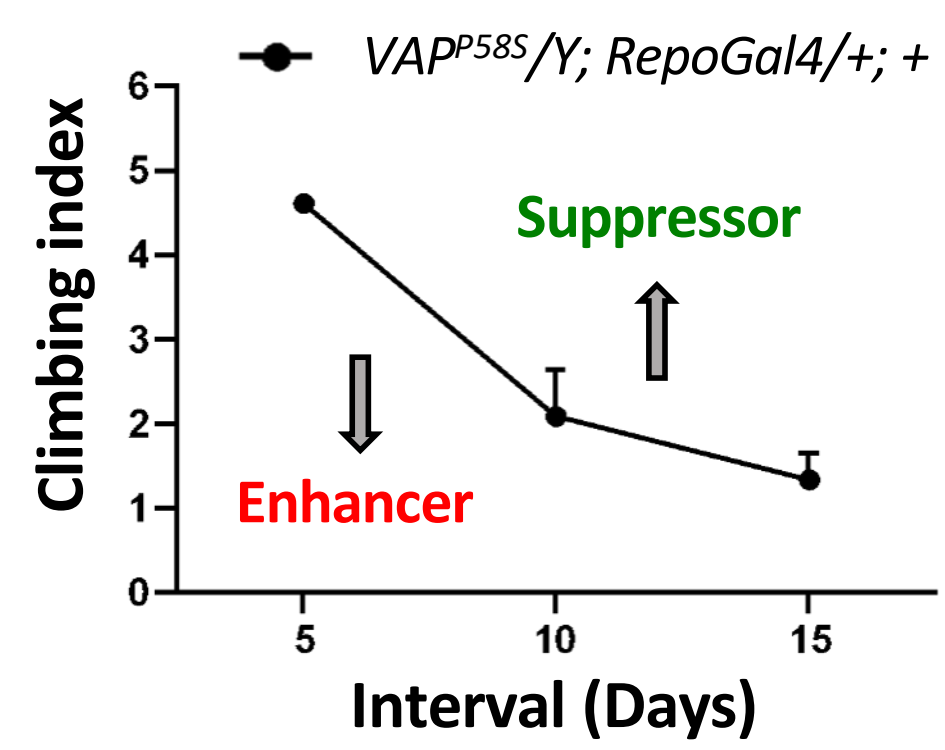

C

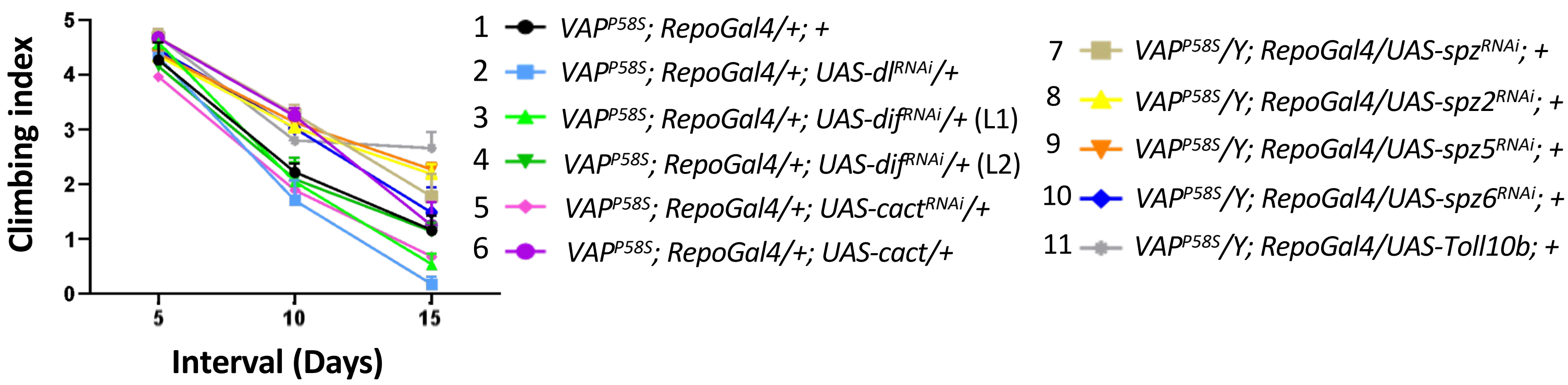

D

	Toll pathway Genotypes	P value for intervals 5	10	15
1	*VAP^{P58S}; RepoGal4/+; +* (Control)			
2	*VAP^{P58S}; RepoGal4/+; UAS-dl^{RNAi}/+*			
3	*VAP^{P58S}; RepoGal4/+; UAS-dif^{RNAi}/+* (L1)			
4	*VAP^{P58S}; RepoGal4/+; UAS-dif^{RNAi}/+* (L2)			
5	*VAP^{P58S}; RepoGal4/+; UAS-cact^{RNAi}/+*			
6	*VAP^{P58S}; RepoGal4/+; UAS-cact/+*			
7	*VAP^{P58S}; RepoGal4/UAS-spz^{RNAi}; +*			
8	*VAP^{P58S}; RepoGal4/UAS-spz2^{RNAi}; +*			
9	*VAP^{P58S}; RepoGal4/UAS-spz5^{RNAi}; +*			
10	*VAP^{P58S}; RepoGal4/UAS-spz6^{RNAi}; +*			
11	*VAP^{P58S}; RepoGal4/UAS-Toll10b; +*			

Suppressor
<0.0001 (****)
<0.001 (***)
<0.01 (**)
<0.05 (*)
>0.05
<0.05 (*)
<0.01 (**)
<0.001 (***)
<0.0001 (****)
Enhancer

E

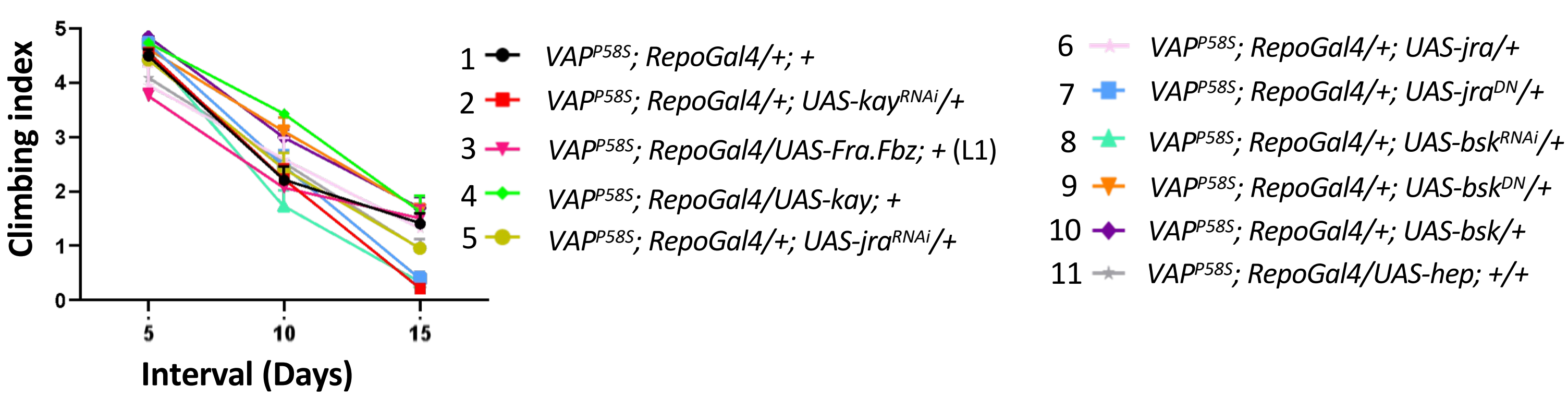

F

	JNK pathway	P value for intervals		
	Genotypes	5	10	15
1	*VAP^{P58S}; RepoGal4/+; +* (Control)			
2	*VAP^{P58S}; RepoGal4/+; UAS-kay^{RNAi}/+*			
3	*VAP^{P58S}; RepoGal4/UAS-Fra.Fbz; +* (L1)			
4	*VAP^{P58S}; RepoGal4/+; UAS-kay/+*			
5	*VAP^{P58S}; RepoGal4/+; UAS-jra^{RNAi}/+*			
6	*VAP^{P58S}; RepoGal4/+; UAS-jra^{WT}/+*			
7	*VAP^{P58S}; RepoGal4/+; UAS-jra^{DN}/+*			
8	*VAP^{P58S}; RepoGal4/+; UAS-bsk^{RNAi}/+*			
9	*VAP^{P58S}; RepoGal4/+; UAS-bsk^{DN}/+*			
10	*VAP^{P58S}; RepoGal4/+; UAS-bsk/+*			
11	*VAP^{P58S}; RepoGal4/UAS-hep; +*			
12	*VAP^{P58S}; RepoGal4/+; UAS-Fra.Fbz/+* (L2)	Lethal		
13	*VAP^{P58S}; RepoGal4/UAS-hep^{CA}; +*	Lethal		

Fig. S8. Enhancer/Suppressor screen in the glia identified *kay* as an important regulator of motor activity in the *VAP^{P58S}* flies

A. Experimental design showing glial knockdown or overexpression of various genes of Toll or JNK pathway using *VAP^{P58S}; RepoGal4; +*.

B. A schematic depicting the effect of gene modulation (knockdown or overexpression) on climbing index. The black curve represents the climbing index for *VAP^{P58S}/Y; RepoGal4/+; +* (control). A leftward shift indicates suppression (improved climbing), whereas a rightward shift indicates enhancement (worsened climbing) of the phenotype. The modulated gene is considered as a "suppressor" or "enhancer" depending on the direction of the shift.

C. Climbing indices for glial knockdown of *dl* (2, light blue), *dif* (L1) (3, light green), *dif* (L2) (4, dark green), *cact* (5, pink), *cact* overexpression (6, purple), knockdown of *spz* (7, brown), *spz2* (8, yellow), *spz5* (9, orange), *spz6* (10, dark blue) and *Toll10b* overexpression (11, grey). *VAP^{P58S}/Y; RepoGal4/+; +* (1, black) has been used as control. Values shown as mean ± SEM. n=25-30, N=3

D. Summary table for the gene modulations in panel (C) with the individual p value for each interval (day 5, 10, 15) depicted using a colour code. Statistical analysis was done by two-way ANOVA. Multiple comparison was done using Tukey's test. *p<0.05, **p<0.01, ***p<0.001, ****p<0.0001

E. Climbing indices for glial knockdown of *kay* (2, red), overexpression of *Fra.Fbz* (3, dark pink), overexpression of *kay* (4, light green), *jra* knockdown (5, ochre), *jra* overexpression (6, light pink), *jra^{DN}* overexpression (7, blue), knockdown of *bsk* (8, sea green), *bsk^{DN}* overexpression (9, orange), *bsk* overexpression (10, purple) and *hep* overexpression (11, grey). *VAP^{P58S}/Y; RepoGal4/+; +* (1, black) has been used as control. Values represented as mean ± SEM. n=25-30, N=3.

F. Summary table for the gene modulations in panel (E) with individual p values being listed. The colour scheme and statistical analysis is as previously described.

Disease Models & Mechanisms • Supplementary information

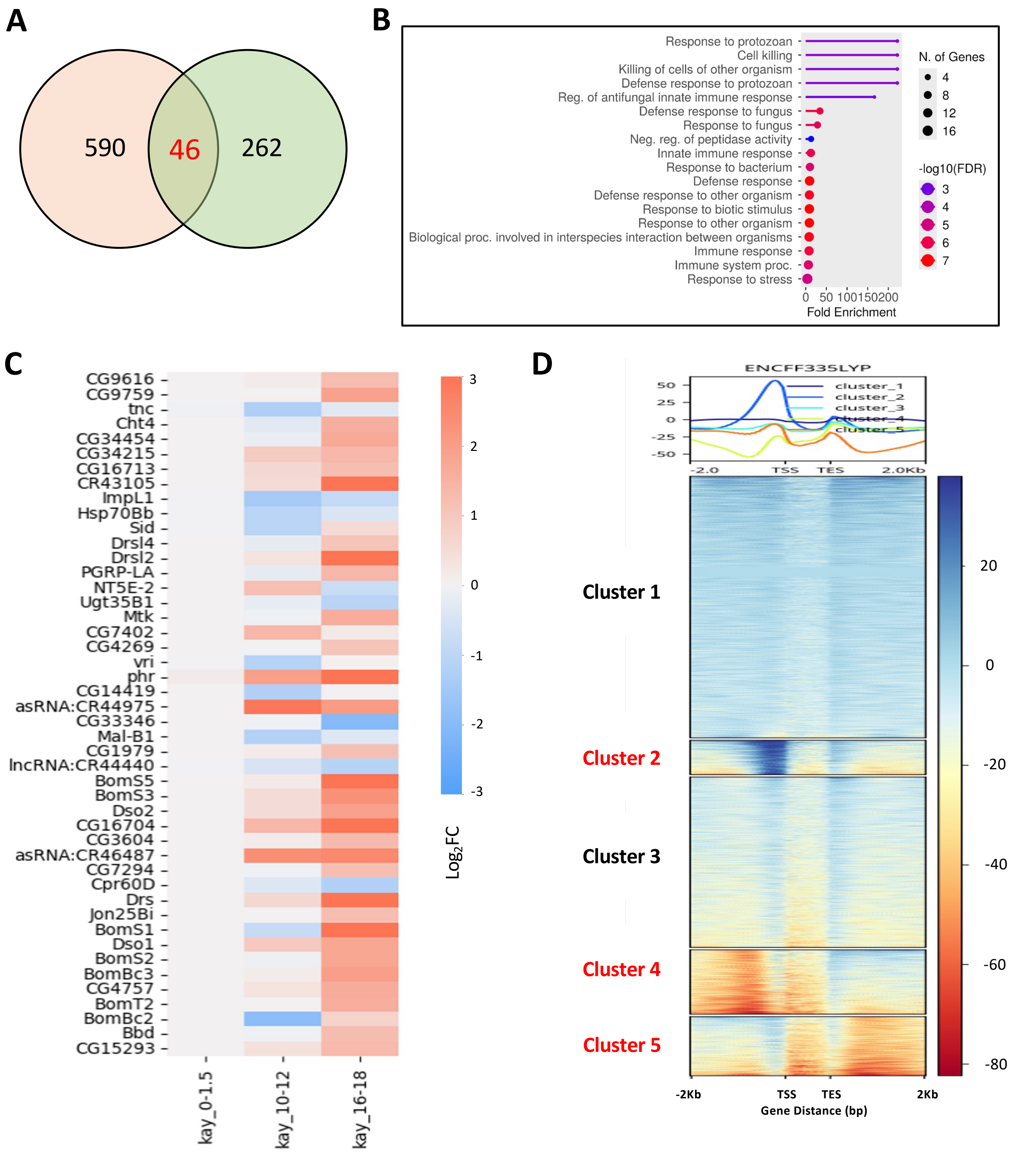

E

Flybase ID	Flybase Symbol	Day	Fold Change (vs VAP^{P58S}; Repo/+; +)		
			2	3	4
FBgn0014018	*Rel*	Day 5	1.42	0.96	0.96
		Day 15	1.30	0.74	0.86
FBgn0260632	*Dl*	Day 5	0.91	0.77	0.82
		Day 15	1.16	0.98	1.07
FBgn0016917	*STAT92E*	Day 5	0.84	0.97	1.01
		Day 15	0.87	0.95	0.82

1: *VAP^{P58S}/Y; RepoGal4/+; +*
2: *VAP^{P58S}/Y; RepoGal4/UAS-Kay^{RNAi}; +*
3: *VAP^{P58S}/Y; RepoGal4/UAS-Kay; +*
4: *VAP^{P58S}/Y; RepoGal4/UAS-Kay^{SCR}; +*

Disease Models & Mechanisms • Supplementary information

Fig. S9. Kay regulates AMPs likely via an indirect mechanism

A. Venn diagram showing overlap between the differentially expressed genes between *VAP*^{P58S} and *VAP*^{WT} (green circle) and the differentially expressed genes upon *kay* knockdown in the embryo (pink circle)

B. Gene Ontology (GO) analysis for the common genes in (A)

C. Heatmap showing the log_2FC values for the common genes in (A) at various timepoints following *kay* knockdown in the embryo

D. Heatmap showing the occupancy of Kay on the gene body of all *Drosophila* genes. Clusters 2, 4 and 5 show genes with enriched binding of Kay. Data extracted from Kay-Chip Seq (ENCSR269HHK) against the input dataset (ENCSR331XEF)

E. Fold change for major immune pathway transcription factors upon glial *kay* modulation in the background of *VAP*^{P58S}

Table S1. Details of the fly lines used in this study

Sr. No.	Line	Genotype	Source	Details
1	VAP^{WT}	VAP^{WT}; +; +	In-house	VAP locus modified using CRISPR /Cas9. No mutation incorporated. Used as control
2	VAP^{P58S}	VAP^{P58S}; +; +	In-house	VAP locus modified using CRISPR/Cas9. VAP^{P58S} mutation incorporated
3	*nos-Cas9*	v^-; *nanos-Cas9*	Gift from Ryu Ueda	
4	VAP^{P58S}; *RepoGal4/CyO*; +	VAP^{P58S}; *RepoGal4/CyO*; +	Balanced in-house	VAP^{P58S} balanced with glial Gal4
5	*RepoGal4*	+; *RepoGal4/CyO*; +	Dr. Bradley Jones, University of Mississippi	Lee et al., 2005
6	$UAS\text{-}dl^{RNAi}$	y[1] sc[*] v[1] sev[21]; P{y[+t7.7] v[+t1.8]=TRiP.HMS00727}attP2	BDSC:32934	
7	$UAS\text{-}dif^{RNAi}$ (L1)	y[1] sc[*] v[1] sev[21]; P{y[+t7.7] v[+t1.8]=TRiP.HM05257}attP2	BDSC:30513	
8	$UAS\text{-}dif^{RNAi}$ (L2)	y[1] v[1]; P{y[+t7.7] v[+t1.8]=TRiP.HM05191}attP2	BDSC:29514	
9	$UAS\text{-}cact^{RNAi}$	y[1] sc[*] v[1] sev[21]; P{y[+t7.7] v[+t1.8]=TRiP.HMS00084}attP2	BDSC:34775	
10	*UAS-cact*	*UASp-cact-eGFP*	Shubha Govind	
11	$UAS\text{-}spz^{RNAi}$	y[1] v[1]; P{y[+t7.7] v[+t1.8]=TRiP.HMJ22258}attP40	BDSC:58499	
12	$UAS\text{-}spz2^{RNAi}$		VDRC:110495	
13	$UAS\text{-}spz5^{RNAi}$	y[1] sc[*] v[1] sev[21]; P{y[+t7.7] v[+t1.8]=TRiP.HMC06330}attP40	BDSC:67229	
14	$UAS\text{-}spz6^{RNAi}$	y[1] sc[*] v[1] sev[21]; P{y[+t7.7] v[+t1.8]=TRiP.HMC04825}attP40	BDSC:57510	
15	*UAS-Toll10b*	P{w[+mC]=UAS-Tl.10b}11, y[1] w[*]	BDSC:58987	Constitutively active Toll

Disease Models & Mechanisms • Supplementary information

16	*UAS-kay^{RNAi}*	y[1] sc[*] v[1] sev[21]; P{y[+t7.7] v[+t1.8]=TRiP.HMS00254}attP2	BDSC:33379	*Drosophila* orthologue of Fos
17	*UAS-Fra.Fbz* (L1)	w[1118]; P{w[+mC]=UAS-Fra.Fbz}5	BDSC:7214	Dominant negative Kay
18	*UAS-Fra.Fbz* (L2)	y[1] w[1118]; P{w[+mC]=UAS-Fra.Fbz}7	BDSC:7215	Dominant negative Kay
19	*UAS-kay* (L1)	w[1118]; P{w[+mC]=UAS-Fra}2	BDCS:7213	
20	*UAS-jra^{RNAi}*	y[1] v[1]; P{y[+t7.7] v[+t1.8]=TRiP.JF01184}attP2	BDSC:31595	*Drosophila* orthologue of Jun
21	*UAS-jra*	y[1] w[1118]; P{w[+mC]=UAS-Jra}2	BDSC:7216	
22	*UAS-jra^{DN}*	y[1] w[1118]; P{w[+mC]=Jbz}10	BDCS:7218	Dominant negative Jra
23	*UAS-bsk^{RNAi}*	y[1] sc[*] v[1] sev[21]; P{y[+t7.7] v[+t1.8]=TRiP.HMS00777}attP2	BDSC:32977	
24	*UAS-bsk^{DN}*	w[*]; P{w[+mC]=UAS-bsk.K53R}20.1a	BDSC:9311	Dominant negative Bsk
25	*UAS-bsk*	w[*]; P{w[+mC]=UAS-bsk.B}2	BDSC:9310	
26	*UAS-hep*	w[*]; P{w[+mC]=UAS-hep.B}2	BDSC:9308	
27	*UAS-hep^{CA}*	w[*]; P{w[+mC]=UAS-Hep.Act}2	BDSC:9306	Constitutively active Hep
28	*UAS-kay* (L2)	*+; UAS-kay^{WT}/CyO; +*	In-house	Inhouse
29	*UAS-kay^{SCR}*	*+; UAS-kay^{SCR}/CyO; +*	In-house	Inhouse-SUMO conjugation resistant form of Kay
30	*w[1118]*		BDSC:3605	

Table S2. Primers used in this study

1	*pGEX-Kay F*	CTGGTTCCGCGTGGATCCCCGGAATTCATGA CGCTGGACAGCTACAAC	Primer pair used to amplify Kay for pGEX-4T1
2	*pGEX-Kay R*	TCGTCAGTCAGTCACGATGCGGCCGCTTATA AGCTGACCAGCTTGGA	
3	*Kay-K357R F*	CTGATGCACATCAGGGACGAGCCACTC	Primer pair used to mutate K357R in Kay
4	*Kay-K357R R*	GAGTGGCTCGTCCCTGATGTGCAT	
5	*pRM-HA-Kay F*	ACGATGTTCCAGATTACGCTGGAGGCGAAAT GACGCTGGACAGCTACAA	Primer pair 28 pRM-HA-Kay R used to amplify Kay for pRM-HA3
6	*pRM-HA-Kay R*	GCAGGTCGACTCTAGAGGATCCTTATAAGCT GACCAGCTTGG	
7	*pUASp-attB-Kay F*	GATCAGATCCGCGGCCGCATGTACCCATACG ATGTTC CAGATTACGCTGGAGGCGGAATGACGCTGG ACAGCTAC AAC	Primer pair to amplify Kay for pRM-HA3
8	*pUASp-attB-Kay R*	TCTAGAGGATCCAGATCCACTAGTTTATAAG CTGACCAG CTTGGAC	
9	*pUASp-attB-HR-Kay F*	CGTTAGGTCCTGTTCATTGGTACCCGCCCGG GGATCAGA TCCGCGGCCGC	Primer pair to amplify pRM HA3 for Kay
10	*pUASp-attB-HR-Kay R*	CGTTAACGTTAACGTTCGAGGTCGACTCTAG AGGATCCA GATCCACTAGT	
11	*rp49 F*	*GACGCTTCAAGGGACAGTATC*	qRT-PCR primer to amplify *rp49*
12	*rp49 R*	*AAACGCGGTTCT GCATGAG*	
13	*CecA1 F*	*CAATCGGAAGCTGGGTG*	qRT-PCR primer to amplify *CecA1*
14	*CecA1 R*	*TAATCATCGG GTCAACCTCGGGC*	
15	*BomBc3 F*	*TCTGATCGGCGCTCATCCC*	qRT-PCR primer to amplify *BomBc3*
16	*BomBc3 R*	*TAGACGGGTTCACCATTCGG*	
17	*BomS5 F*	*TCAGTTTCATTTCAACCGTTGCC*	qRT-PCR primer to amplify *BomS5*
18	*BomS5 R*	*ATTTAACGGAGAAGCCACTGC*	
19	*BomT2 F*	*GGCAGCTGTTAATGCTACGC*	qRT-PCR primer to amplify *BomT2*
20	*BomT2 R*	*GGCTCCAGATGTGAGTGTGT*	
21	*Drs F*	*CTGTCCGGAAGATACAAGGG*	qRT-PCR primer to amplify *Drs*
22	*Drs R*	*TCGCACCAGCACT TCAGACT*	

Dataset 1. Pairwise comparisons of differentially expressed genes for transcriptomic datasets.

Available for download at
https://journals.biologists.com/dmm/article-lookup/doi/10.1242/dmm.052810#supplementary-data

Disease Models & Mechanisms • Supplementary information
